# Repeat Mitral Transcatheter Edge‐to‐Edge Repair for Recurrent Significant Mitral Regurgitation

**DOI:** 10.1161/JAHA.122.028654

**Published:** 2023-04-29

**Authors:** Alon Shechter, Mirae Lee, Danon Kaewkes, Ofir Koren, Sabah Skaf, Tarun Chakravarty, Keita Koseki, Vivek Patel, Raj R. Makkar, Robert J. Siegel

**Affiliations:** ^1^ Department of Cardiology Smidt Heart Institute, Cedars‐Sinai Medical Center Los Angeles CA USA; ^2^ Department of Cardiology Rabin Medical Center Petach Tikva Israel; ^3^ Faculty of Medicine Tel Aviv University Tel Aviv Israel; ^4^ Division of Cardiology, Department of Medicine Samsung Changwon Hospital Changwon Republic of Korea; ^5^ Department of Medicine, Faculty of Medicine Khon Kaen University Thailand; ^6^ Rappaport Faculty of Medicine Technion Israel Institute of Technology Haifa Israel; ^7^ Department of Cardiovascular Medicine The University of Tokyo Tokyo Japan; ^8^ David Geffen School of Medicine University of California Los Angeles Los Angeles CA USA

**Keywords:** MitraClip, mitral transcatheter edge‐to‐edge repair, recurrent mitral regurgitation, transcatheter mitral valve repair, Catheter-Based Coronary and Valvular Interventions

## Abstract

**Background:**

There are limited data on repeat mitral transcatheter edge‐to‐edge repair for recurrent significant mitral regurgitation (MR).

**Methods and Results:**

We conducted a single‐center, retrospective analysis of consecutive patients referred to a second mitral transcatheter edge‐to‐edge repair after a technically successful first procedure. Clinical, laboratory, and echocardiographic measures were assessed up to 1 year after the intervention. The composite of all‐cause death or heart failure (HF) hospitalizations constituted the primary outcome. A total of 52 patients (median age, 81 [interquartile range, 76–87] years, 29 [55.8%] men, 26 [50.0%] with functional MR) met the inclusion criteria. MR recurrences were mostly related to progression of the underlying cardiac pathology. All procedures were technically successful. At 1 year, most patients with available records (n=24; 96.0%) experienced improvement in MR severity or New York Heart Association functional class that was statistically significant but numerically modest. Fourteen (26.9%) patients died or were hospitalized due to HF. These were higher‐risk cases with predominantly functional MR who mostly underwent an urgent procedure and exhibited more severe HF indices before the intervention, as well as an attenuated 1‐month clinical and echocardiographic response. Overall, 1‐year course was comparable to that experienced by patients who underwent only a first transcatheter edge‐to‐edge repair at our institution (n=902). Tricuspid regurgitation of greater than moderate grade was the only baseline parameter to independently predict the primary outcome.

**Conclusions:**

Repeat mitral transcatheter edge‐to‐edge repair is feasible, safe, and clinically effective, especially in non‐functional MR patients without concomitant significant tricuspid regurgitation.

**Nonstandard Abbreviations and Acronyms**
CSMC: Cedars‐Sinai Medical Center
MR: mitral regurgitation
NYHA: New York Heart Association
TEER: transcatheter edge‐to‐edge repair
TR: tricuspid regurgitation

**Clinical Perspective**

**What Is New?**

- Our analysis of 52 real‐world patients demonstrated good feasibility and safety of redo mitral transcatheter edge‐to‐edge repair for recurrent significant mitral regurgitation, as well as improved symptomatic status and regurgitation severity at 1 month and 1 year following the procedure.
- The 1‐year composite rate of all‐cause death or heart failure hospitalizations was 26.9%, closely matching the one observed among patients undergoing transcatheter edge‐to‐edge repair for the first time (n=902).
- A worse combined outcome of death or hospitalizations was associated with functional mitral regurgitation cause and independently predicted by the presence of above‐moderate tricuspid regurgitation at baseline.

**What Are the Clinical Implications?**

- Repeat mitral transcatheter edge‐to‐edge repair is a viable treatment option for patients facing recurrent significant mitral regurgitation after a technically successful first procedure.
- Future, prospective research is needed to validate our findings and attest the added value of our predictive model in the patient selection process.

Mitral transcatheter edge‐to‐edge repair (TEER) is an accepted treatment modality in patients with an above‐moderate native mitral regurgitation (MR) that is accompanied by symptoms or ventricular dysfunction despite guideline‐directed therapy. Among primary MR cases, the procedure results in comparable death rates but lower sustainability of MR reduction compared with surgery,^1^ whereas in functional cases exhibiting heart failure (HF), it demonstrates a superior survival effect when compared with medical treatment alone.^2^ While promising, the performance of mitral TEER in a similarly symptomatic/decompensated, percutaneously intervened valvular disease is less well established.^3^, ^4^, ^5^ Due to patients' elevated risk profile and morphological obstacles imposed by the previously implanted device, redo mitral TEER nevertheless represents 1 of the 2 main treatment options available for recurrent MR following a first mitral TEER, the other being surgical valve replacement^6^, ^7^, ^8^, ^9^ (a third path using percutaneous electrocauterization of the TEER‐induced bridge followed by valve implantation is still experimental^10^). This contrasts recurrent MR after surgery, which may be successfully addressed by surgical^11^, ^12^ or percutaneous^13^, ^14^, ^15^ valve repair or replacement. Considering the widespread use of mitral TEER in developed nations,^16^ the up to 10% recurrence rate of significant MR at 1 year after a technically successful procedure in the real world,^17^ and the setbacks that have been associated with surgical approaches to TEER failure, including a relatively high in‐hospital mortality,^7^ it is important to ascertain the feasibility, safety, and efficacy of repeat mitral TEER. Using a contemporary, large registry, we aimed to explore the characteristics and outcomes of patients undergoing repeat mitral TEER and to identify predictors for adverse prognosis following the reintervention.

## Methods

### Data Availability

The data underlying this article will be shared upon reasonable request to the corresponding author.

### Study Population and Definitions

This is an observational study based on the Cedars‐Sinai Medical Center (CSMC) registry of consecutive mitral TEER procedures performed on adult patients between January 1, 2013, and December 31, 2020. We included patients undergoing redo mitral TEER for above‐moderate MR accompanied by symptoms or ventricular dysfunction despite best tailored medical therapy, after a technically successful first procedure, defined as one that incorporated actual device deployment with no intraprocedural death or reintervention within the first 24 hours.^18^

The primary outcome was the combined rate of all‐cause death or HF hospitalizations at 1 year. Secondary outcomes consisted of individual components of the primary outcome, as well as acute cardiovascular events (including stroke, transient ischemic attack, and myocardial infarction) and any bleeding episodes. All outcomes were defined according to the Mitral Valve Academic Research Consortium criteria.^18^

MR etiologies were determined by intraprocedural transesophageal echocardiographic observations during the first mitral TEER. MR recurrence cause was evaluated using transesophageal echocardiographic images from the repeat procedure. The absence of a definite connection between the existing clip(s) and the mitral leaflet(s) defined a device malfunction. Device‐related factors were further categorized as grasping loss (also referred to as loss of leaflet insertion), clip migration, and leaflet detachment. These have been schematically illustrated by others.^17^, ^19^ Non–device‐related causes for MR recurrence included left atrial (LA) enlargement with resulting annular dilatation and leaflet retraction, left ventricular (LV) remodeling with consequential leaflet tethering, and prolapse/flail progression. Symptomatic status along the study period was reported using the standardized New York Heart Association (NYHA) classification and Kansas City Cardiomyopathy Questionnaire 12 scoring system. Baseline risk assessment used the Society of Thoracic Surgeons (STS) score for mitral valve replacement and the MitraScore.^20^

Our project complied with the Declaration of Helsinki and was approved by the Cedars‐Sinai Institutional Review Board, which waived the need for informed consent.

### Procedural and Echocardiographic Aspects

Mitral TEER was undertaken after a shared decision‐making process and a Heart Team discussion that included at least 1 interventional cardiologist and 1 cardiac surgeon. All used the MitraClip™ system (Abbott Vascular Inc, Santa Clara, CA) and were performed under general anesthesia, via transseptal approach and femoral venous access, and with echocardiographic and fluoroscopic guidance. Monitoring by right heart catheterization was used as well.

Echocardiograms at all stages were performed using the EPIQ ultrasound system (Philips, Andover, MA) and conformed to the relevant American Society of Echocardiography guidelines.^21^, ^22^, ^23^ Two echocardiologists (A.S. and M.L.) blindly reread the studies to ensure data integrity. MR severity was assessed using integration of qualitative and semiquantitative measures. At baseline, it was determined by a preprocedural transthoracic echocardiogram; intraprocedurally, transesophageal echocardiography was used. Global right ventricular function was assessed qualitatively. The pulmonary venous flow pattern was determined on intraprocedural transesophageal echocardiography using the ratio of peak systolic wave velocity to peak diastolic wave velocity, as measured by a pulsed‐wave Doppler beam placed within 1 cm of the pulmonary venous ostia. Pulmonary venous flow pattern improvement was defined as a rise from baseline in the systolic/diastolic velocities ratio on either side, while pulmonary venous flow pattern normalization required the emergence of a ratio of ≥1 on ≥1 side.

### Data Collection and Statistical Analysis

Data were retrieved from an electronic medical chart platform (CS‐Link, Epic, Verona, WI). Per institutional protocol, patients were asked to attend an in‐person appointment at our cardiology outpatient clinic at 1 month and 1 year following the intervention.

Statistical analyses were performed using SPSS version 24 (IBM Corporation, Armonk, NY) and MedCalc version 20.113 (MedCalc, Ostend, Belgium). Variables were reported as frequencies and percentages, medians and interquartile ranges (IQRs), or means and SDs, as appropriate. Comparisons used Pearson's chi‐square, Fisher's exact, Student's *t*, or Mann–Whitney *U* tests. Correlation tests employing Pearson and Spearmen coefficients were undertaken to assess the relationship between variables within the same patient groups. Evaluation of change over time in variables was based on paired‐sample *t*, Wilcoxon, or McNemar tests.

The risk for developing the primary outcome as a function of MR cause, assumed cause of MR recurrence, redo procedure timing, and first procedure location was graphically displayed according to the Kaplan–Meier method, with comparisons of cumulative event‐free survival rates by the log‐rank test. To identify baseline predictors of the primary outcome, and unless specified otherwise, variables of presumed prognostic importance were first analyzed by a univariable Cox regression technique, after which parameters demonstrating a *P* value of <0.05 were integrated into a multivariable analysis model. Harrell's C‐statistic was used to illustrate the discrimination ability of previously studied risk scores for the primary outcome. Finally, repeat procedures were compared with first procedures specifically in patients who have undergone both, or either, at CSMC and whose medical records were retrievable.

Patients with missing values were censored from the relevant analyses in a pairwise fashion. An association was considered statistically significant upon a 2‐sided *P* value of <0.05.

## Results

### Baseline Characteristics of the Study Population

**Figure 1:**
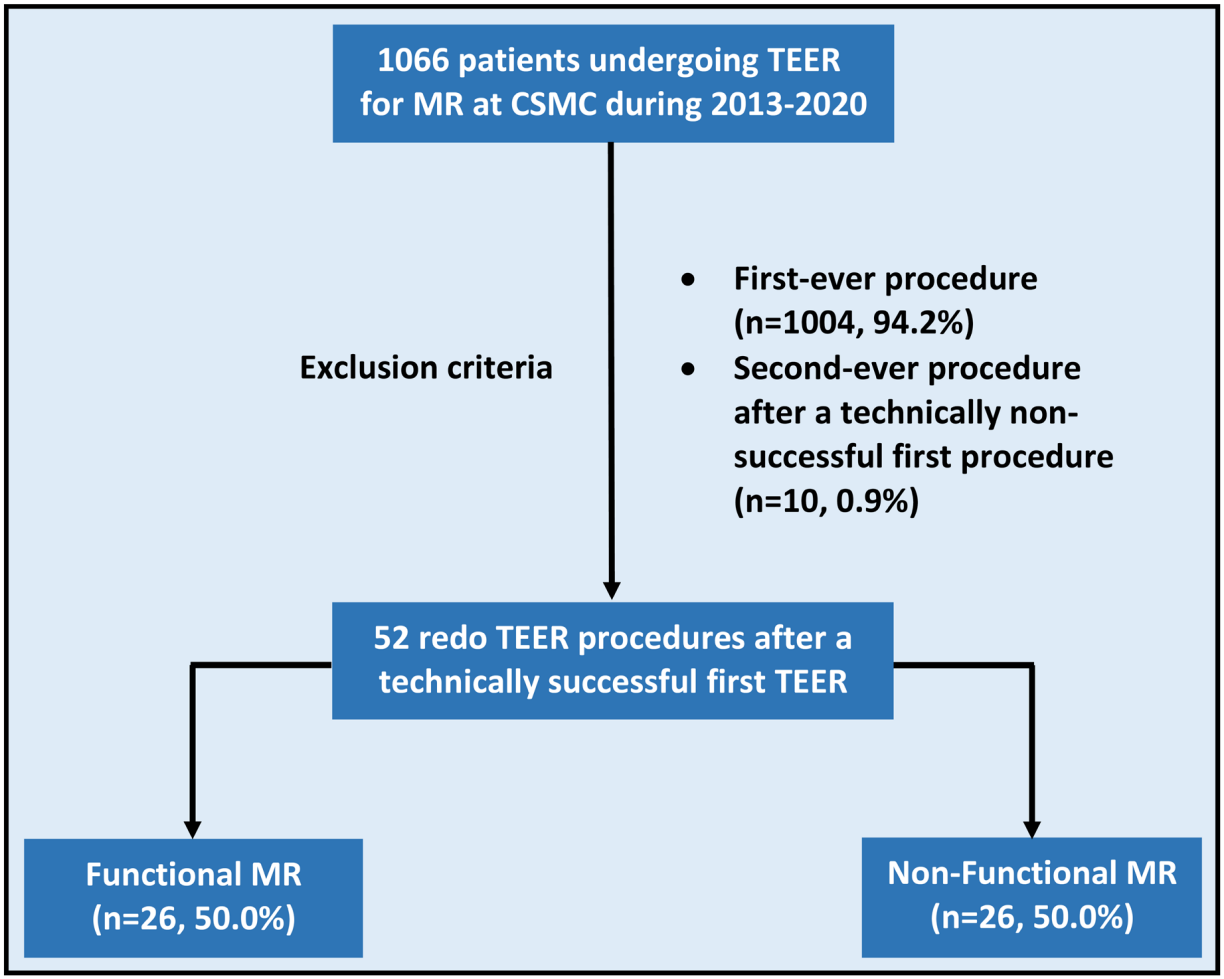
Study flowchart. CSMC indicates Cedars‐Sinai Medical Center; MR, mitral regurgitation; and TEER, transcatheter edge‐to‐edge repair.

Out of 1066 mitral TEER procedures performed at CSMC, 62 (5.8%) were repeat interventions. Of these, 52 (4.9%) followed a technically successful first procedure (Figure 1). Follow‐up duration was 354 (interquartile range, 77–919) days. Baseline and 1‐month echocardiograms were performed 16 (interquartile range, 2–44) days before and 34 (29–42) days after the redo procedure, respectively.

Baseline characteristics of the study cohort are summarized in Table 1. Patients were mostly male (n=29, 55.8%) and elderly (age 81 [interquartile range, 76–87] years) and exhibited a high burden of comorbidities, the most common being hypertension (n=46, 88.5%) and stage ≥III chronic kidney disease (n=38, 74.5%). Notably, they presented to the second TEER with advanced HF, as manifested by a NYHA class IV in 37 (71.2%) cases and a Kansas City Cardiomyopathy Questionnaire 12 score of 53.22 (interquartile range, 24.01–66.32) points. LV ejection fraction was 50% (interquartile range, 26%–62%). These features were reflected in a moderate‐to‐high surgical and percutaneous risk scores.

**Table 1:** Baseline Characteristics of the Study Population

	Redo procedure (N=52)
Demographic details	
Age, y	81 (76–87)
Sex, male	29 (55.8)
Medical conditions	
Noncardiovascular	
Body mass index, kg/m^2^	24.0 (22.0–25.9)
Diabetes	14 (26.9)
Hypertension	46 (88.5)
Smoking	0 (0.0)
Chronic obstructive pulmonary disease	10 (19.2)
Anemia*	30 (57.7)
Stage ≥III chronic kidney disease	38 (74.5)
Cardiovascular	
Previous MI, PCI, or CABG	18 (34.6)
Prior stroke or TIA	7 (13.5)
Peripheral arterial disease	4 (7.7)
Atrial fibrillation/flutter	36 (69.2)
Procedural risk	
STS score for mitral valve repair	6.6 (3.7–13.3)
MitraScore	4 (2–5)
Heart failure features	
New York Heart Association class	
II	3 (5.8)
III	12 (23.1)
IV	37 (71.2)
KCCQ12 score	53.22 (24.01–66.32)
6‐minute walk test distance, m	214 (120–288)
Serum BNP, pg/mL	480 (223–1141)
Mitral regurgitation characteristics	
Mitral regurgitation cause	
Primary	23 (44.2)
Secondary/functional	26 (50.0)
Mixed	3 (5.8)
Mitral regurgitation severity	
Moderate–severe	14 (26.9)
Severe	38 (73.1)
MR PISA EROA, cm^2^	0.37 (0.20–0.50)
MR PISA RVol, mL	31.4 (20.9–67.2)
TMPG, mm Hg	4 (3–5)
Echocardiographic indices	
Left heart	
LV ejection fraction (%)	50 (26–62)
LV end‐diastolic diameter, cm	5.0 (4.6–5.9)
LV end‐systolic diameter, cm	3.6 (3.0–4.7)
LV mass index, ASE formula, g/m^2^	119.7 (93.3–140.8)
Left atrial volume index, cm^3^/m^2^	55.0 (44.2–81.0)
Right heart	
Right ventricular dysfunction	19 (44.2)
Right ventricular diameter, cm	4.2 (3.8–4.8)
Above‐moderate tricuspid regurgitation	19 (37.3)
Right ventricular‐pulmonary arterial coupling	
TAPSE, mm	17 (11–21)
PASP, mm Hg	45 (39–59)
TAPSE/PASP, mm/mm Hg	0.31 (0.27–0.43)
Presentation and preprocedural course	
Acute decompensated heart failure	3 (5.8)
Cardiogenic shock	1 (1.9)
Intravenous inotropic support	3 (5.8)
Mechanical assist device	0 (0.0)
Treatment	
Medications	
Beta blockers	34 (65.4)
RAS inhibitors	26 (50.0)
MRAs	11 (21.2)
Loop diuretics	
Frequency	44 (84.6)
Furosemide‐equivalent dose, mg/day	40 (20–80)
Devices	
CIED	
Total	19 (36.5)
Pacemaker	4 (7.7)
ICD	5 (9.6)
CRT	0 (0.0)
CRTD	10 (19.2)
Hemodialysis	3 (5.8)

Data are presented as number (percentage) or median (interquartile range). ASE indicates American Society of Echocardiography; BNP, B‐type natriuretic peptide; CABG, coronary artery bypass grafting; CIED, cardiac implantable electronic device; CRT, cardiac resynchronization therapy; CRTD, cardiac resynchronization therapy–defibrillator; EROA, effective regurgitant orifice area; ICD, implantable cardioverter defibrillator; KCCQ, Kansas City Cardiomyopathy Questionnaire; LV, left ventricular; MI, myocardial infarction; MR, mitral regurgitation; MRAs, mineralocorticoid receptor antagonists; PASP, pulmonary arterial systolic pressure; PCI, percutaneous coronary intervention; PISA, proximal isovelocity surface area; RAS, renin‐angiotensin system; RVol, regurgitant volume; STS, Society of Thoracic Surgeons; TAPSE, tricuspid annular plane systolic excursion; TIA, transient ischemic attack; and TMPG, transmitral mean pressure gradient.

* Anemia was defined as a blood hemoglobin level of <13 mg/dL in men or <12 mg/dL in women.

**Figure 2:**
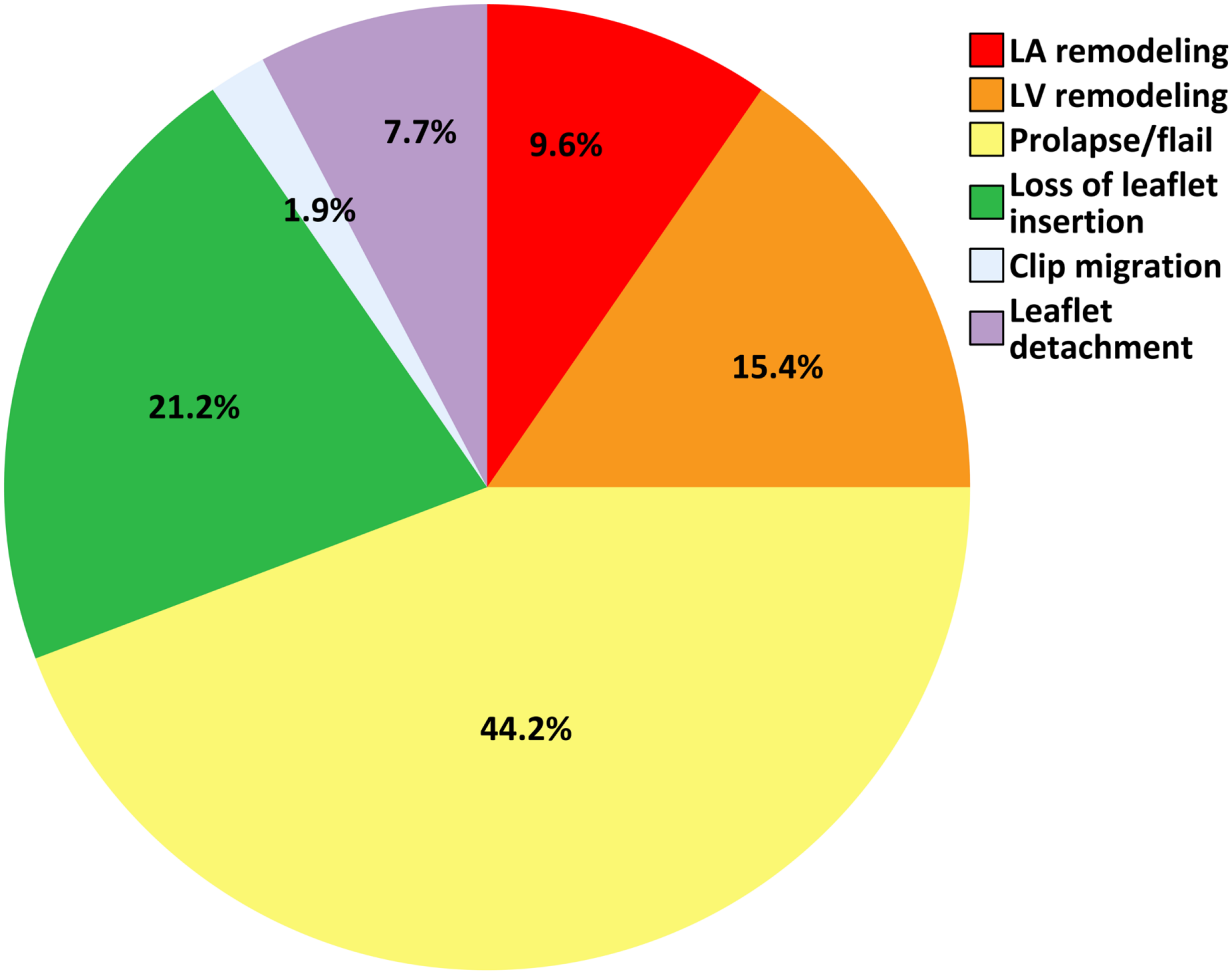
Estimated causes of mitral regurgitation recurrence. LA indicates left atrial; and LV, left ventricular.

Regarding the valvular disease, 26 (50.0%) cases had secondary/functional MR. At the time of the redo intervention, most (n=38, 73.1%) regurgitations were severe. Recurrences were mainly attributed to progression of the underlying disease (Figure 2). LV remodeling causing recurrent MR correlated with functional cause for MR (Spearman's r=0.3; *P*=0.021). Among 39 patients who underwent both first and second TEER at CSMC, MR of less than or equal to mild or less than or equal to moderate degree was immediately achieved after the index procedure in 27 (69.2%) and 38 (97.4%) cases, respectively.

### Procedural Details

Redo mitral TEER was performed at a median of 407 (interquartile range, 129–879) days after the initial intervention; in 23 (44.2%) patients, it was carried out within 1 year. All repeat procedures were concluded by successful device deployment, without any immediate complications or switch to surgery (Table 2). Thirty‐three percent of patients received ≥2 clips, and most (n=42, 80.8%) received second‐ or third‐generation devices. Non‐A2P2 clipping was undertaken in 10 (19.2%) cases and directly correlated with procedural urgency (Spearman's *r*=0.34; *P*=0.013). All but 1 patient were discharged home after a median hospital stay of 1 (interquartile range, 1–6) day.

**Table 2:** Procedural Details and Results

	Redo procedure
	(N=52)
General procedural aspects	
Urgent procedure	14 (26.9)
Total duration, min	111 (80 to 135)
Fluoroscopy duration, min	18 (15 to 29)
Concomitant interventions (ASD closure, TAVR, tricuspid TEER)	0 (0.0)
Conversion to surgery	0 (0.0)
Intraprocedural complications	0 (0.0)
Device parameters	
Clips deployed	
0 (aborted/not deployed)	0 (0.0)
1	35 (67.3)
≥2	17 (32.7)
Median	1 (1 to 2)
Device generation	
First	4 (7.7)
Second	17 (32.7)
Third	25 (48.1)
Fourth	6 (11.5)
Intervention site	
A1P1	2 (3.8)
A2P2	44 (84.6)
A3P3	8 (15.4)
Immediate postprocedural effects	
Echocardiography	
Mitral regurgitation severity mild or less	
After clip deployment	20 (38.5)
At hospital discharge	29 (56.9)
Mitral regurgitation severity moderate or less	
After clip deployment	52 (100.0)
At hospital discharge	49 (96.1)
Transmitral mean pressure gradient, mm Hg	4 (2 to 5)
Pulmonary venous flow pattern*	
Normalization on ≥1 side	28 (60.9)
Improvement on ≥1 side	27 (65.9)
Atrial septal defect	
Before	27 (51.9)
After	52 (100.0)
Right heart catheterization	
V wave, mm Hg	
Before clip deployment	28 (18 to 39)
After clip deployment	25 (19 to 33)
*P* value for change	0.027^†^
Mean left atrial pressure, mm Hg	
Before clip deployment	18 (14 to 25)
After clip deployment	19 (12 to 23)
Delta	−3 (−9 to 4)
*P* value for change	0.14
Mean pulmonary arterial pressure, mm Hg	
Before clip deployment	33 (25 to 46)
After clip deployment	28 (21 to 36)
*P* value for change	0.20
Postprocedural course	
Intensive care unit stay duration, h	4.1±19.2
Hospitalization length, d	1 (1 to 6)
Discharge home	51 (98.1)

Data are presented as number (percentage), median (interquartile range), or mean±SD. ASD indicates atrial septal defect; TAVR, transcatheter aortic valve replacement; and TEER, transcatheter edge‐to‐edge repair.

* Improvement and normalization of the pulmonary venous flow pattern were defined as a delta systolic/diastolic velocities ratio of >1 and as a postprocedural systolic/diastolic velocities ratio of ≥1, respectively.

### Postprocedural Regurgitation Severity and Heart Failure Indices

A reduction in MR severity to mild or less was observed in 20 (38.5%) patients immediately after clip deployment and in 29 (55.8%) patients by the time of hospital discharge (Table 2); up to moderate MR was observed in 52 (100.0%) and 49 (96.1%) cases, respectively. Intraprocedural improvement in pulmonary venous flow pattern was recorded in 27 (65.9%) procedures. While a significant drop in the LA V wave was recorded, the mean LA pressure, reported in 32 patients, was unchanged. Atrial septal defect, present before the septal puncture in 27 (51.9%) patients (12/19 with and 15/32 without significant tricuspid regurgitation [TR]; *P*=0.26), was universally observed after clip deployment. At both stages, mild left‐to‐right shunt was noted in all but 2 patients who developed at the postprocedure phase a mild bidirectional shunt.

One‐month and 1‐year MR severity was reported in 37 (71.2%) and 20 (38.5%) patients, respectively. The corresponding figures regarding functional status were 41 (78.8%) and 23 (44.2%), respectively. A total of 25 (48.1%) patients had records on MR grade or NYHA class at 1 year.

**Figure 3:**
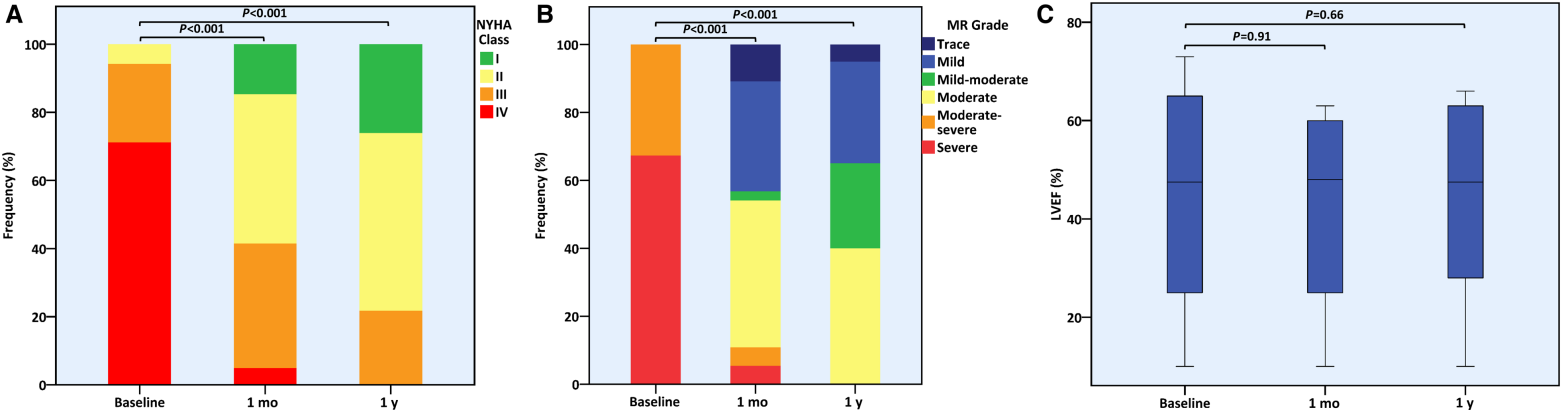
Transcatheter edge‐to‐edge repair for recurrent significant mitral regurgitation. **A**, Functional status; **B**, mitral regurgitation severity; **C**, left ventricular ejection fraction. Repeat mitral transcatheter edge‐to‐edge repair for recurrent significant mitral regurgitation led to significant improvement in functional status (**A**) and regurgitation severity (**B**), both at 1 month and 1 year. Left ventricular ejection fraction was not changed from baseline (**C**). LVEF indicates left ventricular ejection fraction; MR, mitral regurgitation; and NYHA, New York Heart Association.

Most patients with available data exhibited a significant improvement in functional status and MR severity both at the 1‐month and 1‐year marks, such that by 1 year, NYHA class decreased in 21 (91.3%) and MR regressed in 20 (100.0%) cases compared with baseline (Table 3 and Figure 3). While mildly symptomatic HF (ie, NYHA class I–II) or up to moderate MR was present in 24 (96.0%) patients with available records by the end of the first postprocedural year (mildly symptomatic HF, n=18, 78.3%; up to moderate MR, n=20, 100.0%), the combination of both was observed in 14 patients (73.7%). No significant differences compared with baseline were seen in loop diuretic dosage, serum BNP (B‐type natriuretic peptide) levels, or echocardiographic measures of cardiac function and dimensions.

**Table 3:** One‐Month and 1‐Year Heart Failure and Mitral Regurgitation Indices

	1 mo	1 y
Clinical		
NYHA class		
I–II	24 (58.5)	18 (78.3)
Change from baseline (classes)	−1.3±0.7	−1.5±0.8
*P* value vs baseline	<0.001*	<0.001*
Improved (reduced) from baseline	36 (87.8)	21 (91.3)
Furosemide‐equivalent dose		
Median, mg/d	40 (0 to 80)	40 (20 to 80)
Change from baseline, mg/d	0 (−20 to 0)	0 (0 to 20)
*P* value vs baseline	0.49	0.14
Improved (reduced) from baseline	16 (33.3)	2 (10.5)
Laboratory		
Serum B‐type natriuretic peptide		
Median, pg/mL	510 (227 to 1137)	524 (271 to 1063)
Change from baseline, pg/mL	36 (−156 to 334)	65 (−11 to 331)
*P* value vs baseline	0.23	0.13
Improved (reduced) from baseline	9 (31.0)	4 (30.8)
Echocardiographic		
Mitral regurgitation severity		
Mild or less	16 (43.2)	7 (35.0)
*P* value vs baseline	<0.001*	0.029*
Moderate or less	33 (89.2)	20 (100.0)
*P* value vs baseline	<0.001*	<0.001*
Improved from baseline	33 (89.2)	20 (100.0)
Transmitral mean pressure gradient		
Median, mm Hg	5 (3 to 7)	4 (2 to 5)
Change from baseline, mm Hg	1 (−1 to 3)	−1 (−1 to 2)
*P* value vs baseline	0.07	0.96
Left ventricular ejection fraction		
Median, %	56 (32 to 61)	55 (30 to 60)
Change from baseline, %	1 (−5 to 5)	0 (−9 to 13)
*P* value vs baseline	0.91	0.66
Improved (increased) from baseline	18 (51.4)	9 (45.0)
Left ventricular end‐systolic diameter		
Median, cm	3.5 (2.7 to 4.4)	3.8 (2.9 to 5.3)
Change from baseline, cm	−0.1 (−0.4 to 0.3)	0.0 (−0.1 to 0.4)
*P* value vs baseline	0.58	0.47
Improved (reduced) from baseline	16 (59.3)	6 (40.0)
Left atrial volume index		
Median, cm^3^/m^2^	61.9 (41.9 to 80.0)	54.0 (39.9 to 76.1)
Change from baseline, cm^3^/m^2^	−2.0 (−10.1 to 15.8)	−55.5 (−81.0 to 41.5)
*P* value vs baseline	0.39	0.27
Improved (reduced) from baseline	8 (66.7)	27 (96.4)
Pulmonary arterial systolic pressure		
Median, mm Hg	48 (37 to 56)	45 (35 to 56)
Change from baseline, mm Hg	6 (−10 to 12)	−5 (−16 to 11)
*P* value vs baseline	0.40	0.67
Improved (reduced) from baseline	8 (40.0)	7 (63.6)
TR severity above‐moderate	14 (40.0)	6 (30.0)
*P* value vs baseline	0.73	1.00
Combined		
NYHA class I–II and MR severity mild or less	9 (24.3)	5 (25.0)
*P* value vs baseline	<0.001*	<0.001*
NYHA class I–II and MR severity moderate or less	20 (54.1)	14 (73.7)
*P* value vs baseline	<0.001*	<0.001*

Data are presented as number (percentage), median (interquartile range), or mean±SD. MR indicates mitral regurgitation; NYHA, New York Heart Association; and TR, tricuspid regurgitation.

* Denotes statistical significance.

### Clinical Outcomes

Overall, 14 (26.9%) patients experienced the primary outcome, a composite of all‐cause death (n=9, 17.3%) or HF hospitalizations (n=7, 13.5%) during the first year after repeat mitral TEER (Table 4). Most events (n=12, 85.7%) took place >1 month after the procedure. Acute cardiovascular events (n=2, 3.8%) and Mitral Valve Academic Research Consortium bleeding episodes (n=2, 3.8%) were uncommon. Of the 3 mitral reinterventions undertaken after the second procedure, 2 were third‐time mitral TEER (on days 41 and 49), and 1 was a surgical valve replacement (on day 49). There were no subsequent atrial septal defect closures during the follow‐up period.

**Table 4:** Clinical Outcomes

	Frequency		Event‐free survival time at 1 y (d)
	1 mo	1 y	
Primary outcome			
All‐cause death or heart failure hospitalizations	2 (3.8)	14 (26.9)	219.6±152.9
Secondary outcomes			
All‐cause death	0 (0.0)	9 (17.3)	238.7±145.0
Heart failure hospitalizations	2 (3.8)	7 (13.5)	219.6±152.9
Mitral reintervention	0 (0.0)	3 (5.8)	224.6±150.1
MI/Stroke/TIA	1 (1.9)	2 (3.8)	46.5±20.8
MVARC bleeding	1 (1.9)	2 (3.8)	130.0±154.1

Data are presented as number (percentage) or mean±standard deviation. MI indicates myocardial infarction; MVARC, Mitral Valve Academic Research Consortium; and TIA, transient ischemic attack.

### Prediction of the Primary Outcome

The 14 patients who sustained the combined end point of all‐cause death or HF hospitalizations presented to the redo procedure with more pronounced HF and right‐sided echocardiographic abnormalities (including significant TR), a higher procedural risk, almost twice the prevalence of functional MR, and a numerically higher burden of preexistent atrial septal defect compared with the 38 event‐free cases (Tables S1 and S2). Their reinterventions were performed earlier and mostly on an urgent basis (ie, nonelective or unplanned) and used a nonsignificantly higher number of clips (Table S2). Following the procedure, patients who experienced the primary outcome were less likely to achieve an immediate reduction in MR to less than or equal to mild, as well as symptomatic relief at 1 month (Tables S2 and S3). Numerically fewer patients in this group also maintained an up to mild MR at discharge and at 1 month. No differences were demonstrated between patients who did and did not experience the primary outcome in regard to the estimated cause of MR recurrence, baseline LV function and diameters, periprocedural values of the mean LA pressure, rates of up to moderate MR at all time points explored, and the availability of 1‐month data on MR grade and NYHA class. Notably, all 3 patients who underwent a repeat TEER within the first month of the initial procedure, and most (5/8, 62.5%) of those in whom the A3P3 segment was targeted, sustained the primary outcome. Also, all 14 patients who died or were hospitalized due to HF by 1 year following the redo procedure remained in NYHA class of >II or had greater than mild residual MR after the first month.

**Figure 4:**
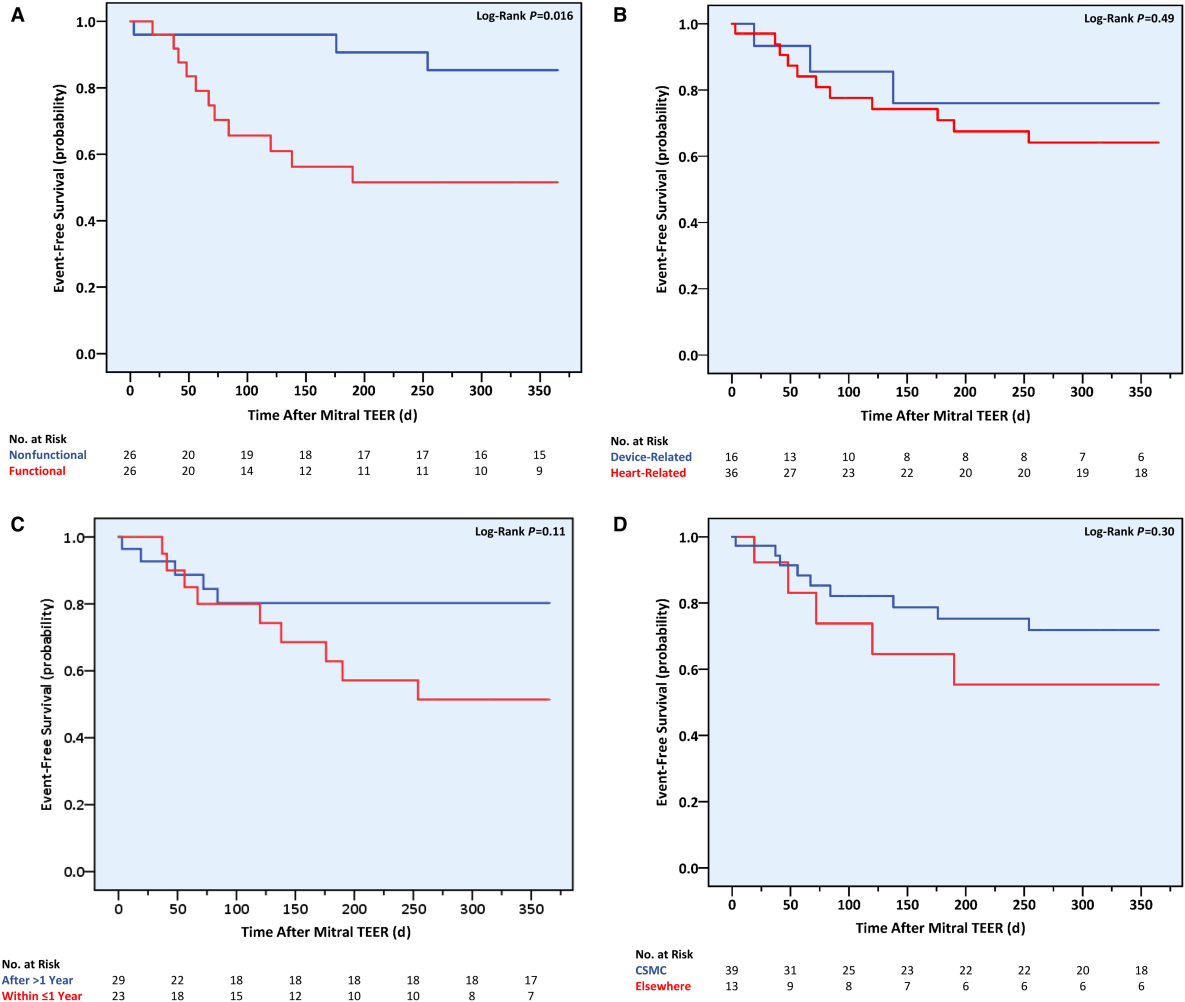
One‐year cumulative incidence of all‐cause death or heart failure hospitalizations following repeat mitral transcatheter edge‐to‐edge repair according to baseline mitral regurgitation cause, recurrent mitral regurgitation cause, redo procedure timing, and first procedure institution. **A**, Baseline mitral regurgitation cause; **B**, recurrent mitral regurgitation cause; **C**, redo procedure timing in relation to first procedure; **D**, first procedure institution. CSMC indicates Cedars‐Sinai Medical Center; and TEER, transcatheter edge‐to‐edge repair.

**Figure 5:**
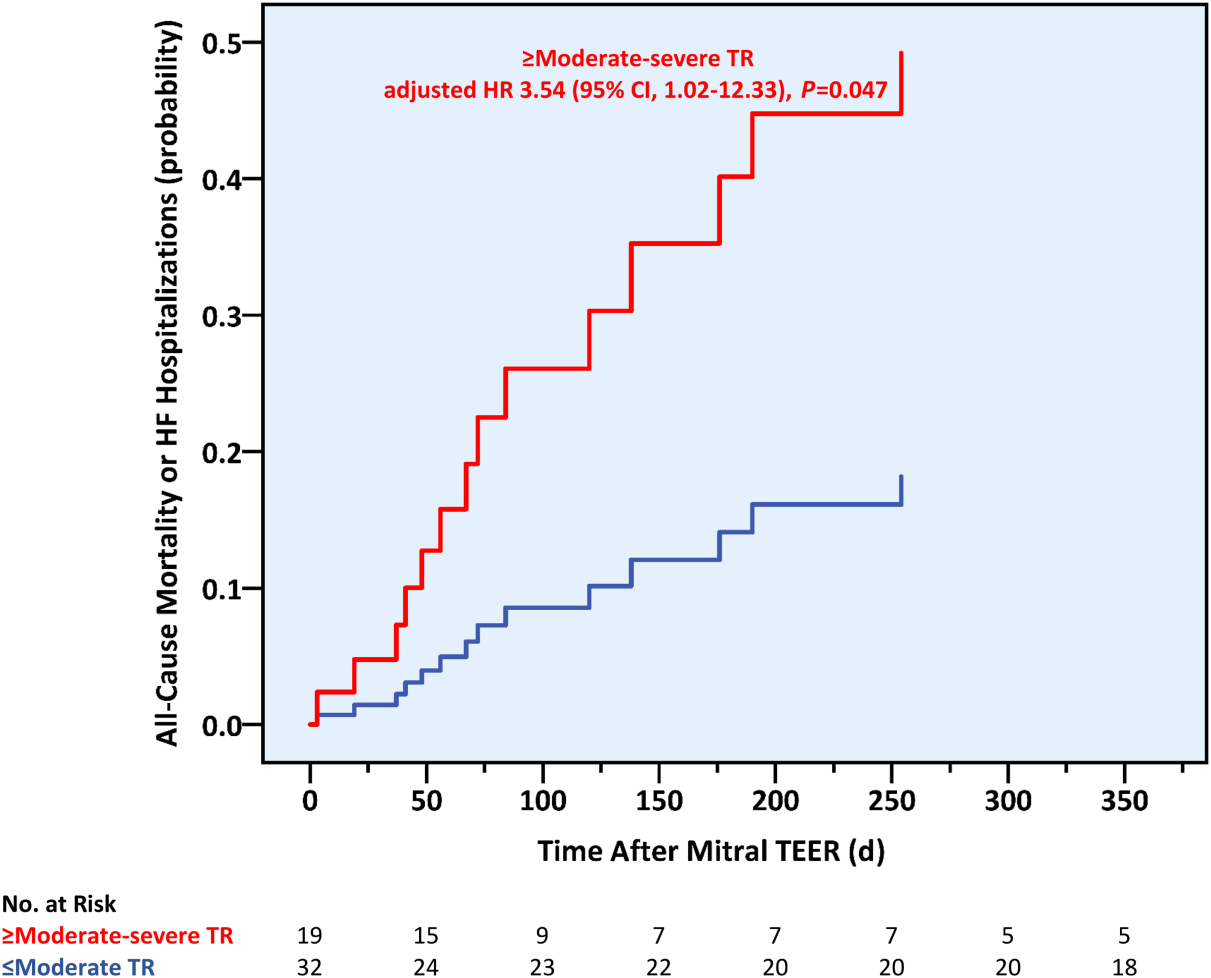
Probability of 1‐year combined outcome of all‐cause death or heart failure hospitalizations according to baseline tricuspid regurgitation severity. HF indicates heart failure; HR, hazard ratio; TEER, transcatheter edge‐to‐edge repair; and TR, tricuspid regurgitation.

**Figure 6:**
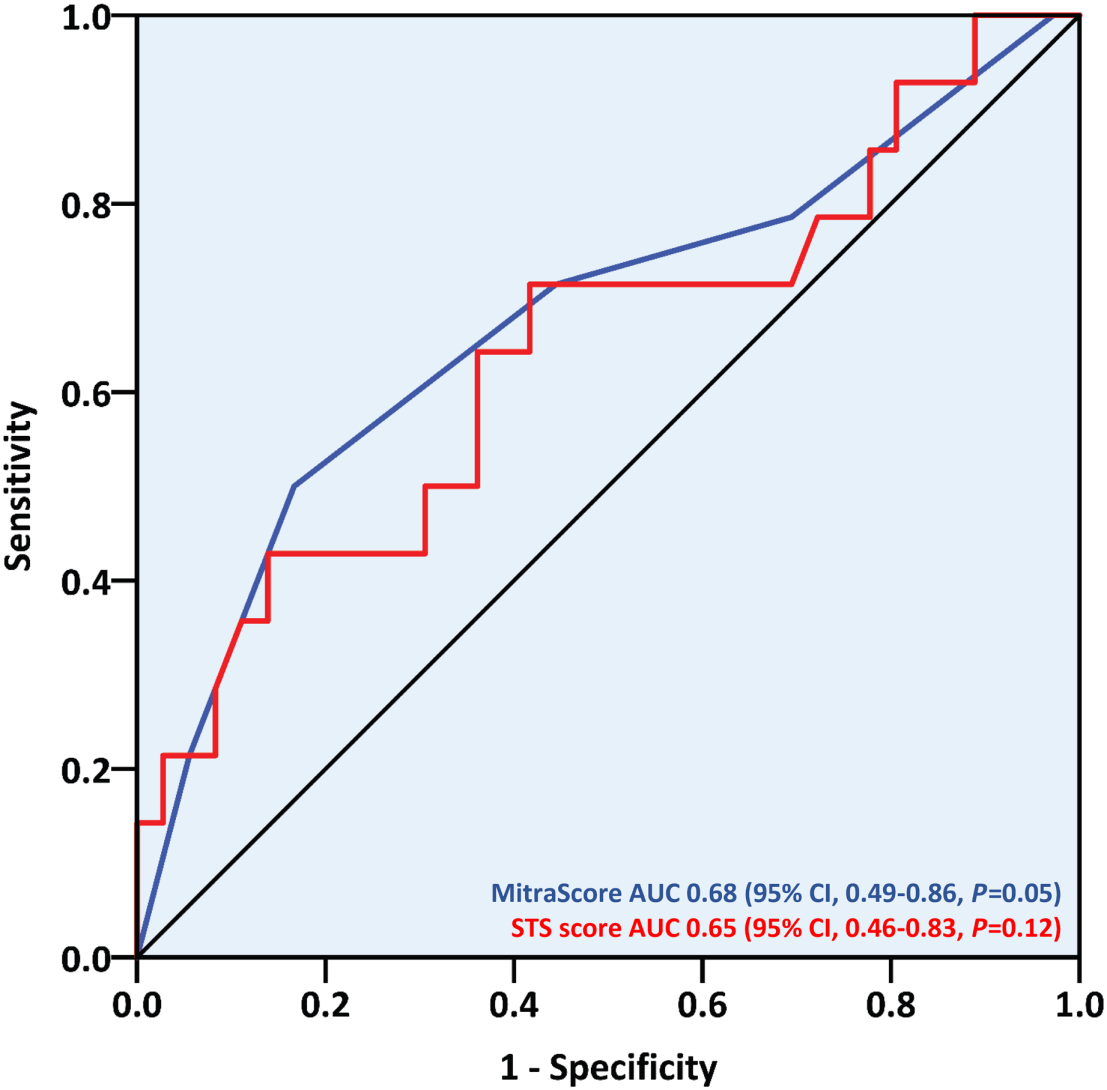
C‐statistic values of risk models for the combined outcome of all‐cause death or heart failure hospitalizations following redo mitral transcatheter edge‐to‐edge repair. AUC indicates area under the curve; and STS, Society of Thoracic Surgeons.

Using Kaplan–Meier analysis, a higher 1‐year cumulative event rate was observed in patients with functional versus nonfunctional MR (42.3% versus 11.5%; log‐rank *P*=0.016) (Figure 4). By contrast, no differences in event‐free survival times were shown when considering the recurrence cause, the redo procedure's timing (≤1 versus >1 year after the initial procedure), or the first procedure's location (CSMC versus non‐CSMC). After multivariable analysis, above‐moderate TR emerged as the sole independent predictor of the primary outcome, more than tripling its risk (hazard ratio [HR], 3.54 [95% CI, 1.02–12.33]; *P*=0.047) (Table 5 and Figure 5). This echocardiographic parameter affected 10 of the 14 primary outcome cases, and patients with significant TR suffered higher rates of all‐cause death (7/19 [36.8%] versus 2/32 [6.3%]; *P*=0.009), HF hospitalizations (5/19 [26.3%] versus 2/32 [6.3%]; *P*=0.044) and the composite of both (10/19 [52.6%] versus 4/32 [12.5%]; *P*=0.002) during the first postprocedural year. Neither the Society of Thoracic Surgeons score for mitral valve repair nor the MitraScore demonstrated discriminative capabilities for the primary outcome (Figure 6).

**Table 5:** Cox Proportional Hazard Model for the Combined Outcome of All‐Cause Mortality or Heart Failure Hospitalizations at 1 Year

	Univariable		Multivariable	
	HR (95% CI)	*P* value	HR (95% CI)	*P* value
Baseline clinical characteristics				
Age (continuous)	0.98 (0.91–1.06)	0.65		
Sex, male	0.49 (0.17–1.42)	0.19		
Body mass index (continuous)	0.97 (0.83–1.14)	0.97		
Diabetes	0.93 (0.26–3.33)	0.91		
Hypertension	0.61 (0.22–12.95)	0.61		
Chronic obstructive pulmonary disease	1.30 (0.36–4.68)	0.68		
Anemia*	2.15 (0.67–6.85)	0.20		
Stage ≥III chronic kidney disease	4.09 (0.53–31.34)	0.18		
Previous MI, PCI, or CABG	1.99 (0.69–5.72)	0.20		
Prior stroke or TIA	0.47 (0.06–3.56)	0.46		
Peripheral arterial disease	1.23 (0.16–9.45)	0.84		
Atrial fibrillation/flutter	0.41 (0.14–1.17)	0.10		
No CIED	0.33 (0.11–0.99)	0.050		
New York Heart Association class IV	4.84 (0.63–36.06)	0.13		
KCCQ12 score (continuous)	0.99 (0.95–1.04)	0.74		
6‐minute walk distance (continuous)	0.97 (0.91–1.04)	0.39		
Furosemide‐equivalent daily dose (continuous)	1.01 (0.99–1.02)	0.20		
Serum BNP (continuous)†	1.004 (1.0002–1.01)	0.036		
No use of beta blockers	1.05 (0.35–3.14)	0.93		
No use of RAS inhibitors	1.31 (0.46–3.79)	0.61		
No use of MRAs	0.56 (0.19–1.69)	0.31		
Baseline echocardiographic and hemodynamic parameters				
Functional mitral regurgitation	4.27 (1.19–15.35)	0.026		
Severe mitral regurgitation	1.25 (0.42–3.74)	0.69		
Transmitral mean pressure gradient (continuous)	0.74 (0.53–1.02)	0.17		
LV ejection fraction (continuous)	0.99 (0.96–1.02)	0.40		
LV end‐diastolic diameter (continuous)	1.36 (0.88–2.09)	0.16		
LV end‐systolic diameter (continuous)	1.26 (0.88–1.79)	0.21		
Left atrial volume index (continuous)	1.04 (0.98–1.03)	0.80		
Right ventricular dysfunction	2.72 (0.86–8.58)	0.09		
Above‐moderate tricuspid regurgitation§	5.25 (1.63–16.87)	0.005	3.54 (1.02–12.33)	0.047¶
			4.22 (1.29–13.85)	0.017‖ ^,^ ¶
Pulmonary arterial systolic pressure (continuous)	0.98 (0.92–1.03)	0.37		
TAPSE/PASP (continuous)	0.15 (0.01–24.31)	0.46		
Atrial septal defect	2.29 (0.72–7.30)	0.16		
V wave (continuous)	1.02 (0.97–1.06)	0.48		
Mean left atrial pressure (continuous)	1.02 (0.95–1.11)	0.56		
Mean right atrial pressure (continuous)	1.04 (0.88–1.23)	0.63		
Mean pulmonary arterial pressure (continuous)	0.99 (0.93–1.05)	0.66		
Presentation to procedure				
Acute decompensated heart failure	1.10 (0.14–8.44)	0.93		
Cardiogenic shock	3.35 (0.43–26.03)	0.25		
Assumed mitral regurgitation recurrence cause				
Cardiac remodeling				
Left atrial remodeling	0.57 (0.08–4.37)	0.59		
LV remodeling	3.91 (1.22–12.57)	0.022		
Left atrial or LV remodeling	2.06 (0.69–6.15)	0.20		
Prolapse/flail progression	0.78 (0.27–2.26)	0.65		
Device‐related issues	0.64 (0.18–2.30)	0.50		
Loss of leaflet insertion	1.12 (0.31–4.00)	0.87		
Clip migration	0.05 (0.01–732.80)	0.68		
Leaflet detachment	0.04 (0.01–304.34)	0.49		
Functional MR or LV remodeling causing recurrent MR‡	4.27 (1.19–15.35)	0.026	3.02 (0.79–11.59)	0.11
			3.49 (0.95–12.84)	0.16‖
Time from first procedure				
Continuous	0.99 (0.99–1.01)	0.08		
≤1 mo	9.31 (2.38–36.70)	0.001	3.17 (0.75–13.51)	0.12
≤1 y	2.40 (0.80–7.19)	0.12		
First procedure not at Cedars‐Sinai Medical Center	1.16 (0.32–4.14)	0.83		
Postprocedural data availability				
Absence of 1‐mo data on MR severity	2.35 (0.81–6.78)	0.11		
Absence of 1‐mo data on NYHA class	2.09 (0.65–6.67)	0.22		

CABG indicates coronary artery bypass grafting; CIED, cardiac implantable electronic device; CKD, chronic kidney disease; HR, hazard ratio; KCCQ, Kansas City Cardiomyopathy Questionnaire; LV, left ventricular; MR, mitral regurgitation; MRAs, mineralocorticoid receptor antagonists; NYHA, New York Heart Association; PCI, percutaneous coronary intervention; RAS, renin‐angiotensin system; TAPSE, tricuspid annular plane systolic excursion; TIA, transient ischemic attack; and TR, tricuspid regurgitation.

* Anemia was defined as a blood hemoglobin level <13 mg/dL in men or 12 mg/dL in women.

^†^ Serum BNP correlated with significant TR (Pearson's r=0.4; *P*=0.009), functional MR/LV remodeling causing recurrent MR (Pearson's r=0.3, *P*=0.017), and very early redo procedure (Pearson's r=0.3, *P*=0.037) and was therefore omitted from the multivariable analysis.

^‡^ As functional MR and LV remodeling causing recurrent MR were significantly associated and correlated (Spearman's r=0.3; *P*=0.021), they were combined into a single variable.

^§^ Significant TR remained the sole variable to exhibit a significant association with the primary outcome also upon inclusion, in the multivariable analysis, of parameters with a *P* value of <0.1 on univariate analyses (ie, atrial fibrillation, no use of CIED, and right ventricular dysfunction).

^‖^ Multivariable analysis was repeated without the ≤1‐month redo TEER variable, as redo TEER was performed within 1 month of the first procedure in 3 patients.

^¶^ Denotes statistical significance in the multivariable model.

### Functional Versus Nonfunctional Mitral Regurgitation

Patients who underwent redo TEER with functional MR suffered a more advanced HF at baseline and were more likely to have an above‐moderate TR and to undergo an urgent procedure (Tables S4 and S5). Their short‐term biochemical and echocardiographic response was attenuated, with only 7 (26.9%) patients demonstrating an immediate reduction in MR severity to less than or equal to mild (Table S5). One‐month and 1‐year MR grade and functional status, however, were comparable to those observed in nonfunctional MR cases (Table S6). An overall higher clinical event rate was experienced by the functional MR subgroup, reaching statistical significance for death (Table S7).

### Redo Versus First Procedures Performed at CSMC on the Same Patients

Thirty‐nine patients were identified who underwent both the first and redo mitral TEER procedures at CSMC and for whom there were available records. Presentation of these patients to the second procedure was characterized by a worse HF status, signified by a higher prevalence of NYHA functional class IV and lower LV ejection fraction and right ventricular pulmonary arterial coupling values, as well as a trend toward a worse procedural risk (Table S8). Interestingly, MR was reported less commonly as severe before the redo procedure. Compared with the index intervention, repeat TEER used newer‐generation devices and numerically fewer clips and targeted non‐A2P2 segments more commonly (Table S9). No differences were noted in total and fluoroscopy times and regarding intraprocedural complications. A summary of baseline and periprocedural echocardiographic and hemodynamic parameters observed in the 2 procedures is provided in Table 6.

**Table 6:** Selected Baseline and Periprocedural Echocardiographic and Hemodynamic Parameters Observed in First and Redo Procedures Performed at Cedars‐Sinai

	First procedure (N=39)	Redo procedure (N=39)	*P* value
Baseline mitral regurgitation characteristics			
Mitral regurgitation severity			0.018†
Moderate–severe	1 (2.6)	10 (25.6)	
Severe	38 (97.4)	29 (74.4)	
TMPG, mm Hg	2 (2–3)	4 (3–5)	0.001†
Immediate postprocedural echocardiographic parameters			
Mitral regurgitation severity			
Mild or less	27 (69.2)	14 (35.9)	0.003†
Moderate or less	38 (97.4)	39 (100.0)	1.00
TMPG, mm Hg	3 (2–4)	3 (2–5)	0.15
PVFP*			
Normalization on ≥1 side	20 (54.1)	21 (60.0)	0.61
Improvement on ≥1 side	26 (76.5)	20 (64.5)	0.29
Periprocedural right heart catheterization parameters			
V wave, mm Hg			
Before	30 (20–40)	28 (18–38)	0.83
After	21 (13–26)	26 (18–34)	0.048†
*P* value for change	0.001†	0.045†	NA
Mean LA pressure, mm Hg			
Before	18 (13–23)	17 (14–27)	0.80
After	14 (10–19)	19 (13–24)	0.016†
*P* value for change	0.003†	0.19	NA
Mean PA pressure, mm Hg			
Before	27 (23–30)	34 (23–47)	0.044†
After	25 (22–30)	28 (21–38)	0.91
*P* value for change	0.51	0.26	NA

Data are presented as number (percentage) or median (interquartile range). LA indicates left atrial; NA, not applicable; PA, pulmonary arterial; PVFP, pulmonary venous flow pattern; and TMPG, transmitral mean pressure gradient.

* Improvement and normalization of the pulmonary venous flow pattern were defined as a delta *S*/*D* velocities ratio of >1 and as a postprocedural *S*/*D* velocities ratio of ≥1, respectively.

^†^ Denotes statistical significance.

Focusing on 37 patients in whom the 2 procedures were separated by more than a month, similar 1‐month effects were noted in terms of absolute and relative (compared with baseline) clinical and laboratory indices of HF, as well as comparable rates of reduction of MR severity to less than or equal to mild or less than or equal to moderate—this is despite significantly lower rates of less than or equal to mild MR immediately after clip deployment and at discharge time and a numerically higher rate of 1‐month significant TR after the redo procedure (Tables S9 and S10).

### All Redo Versus All First Procedures Performed at CSMC

**Figure 7:**
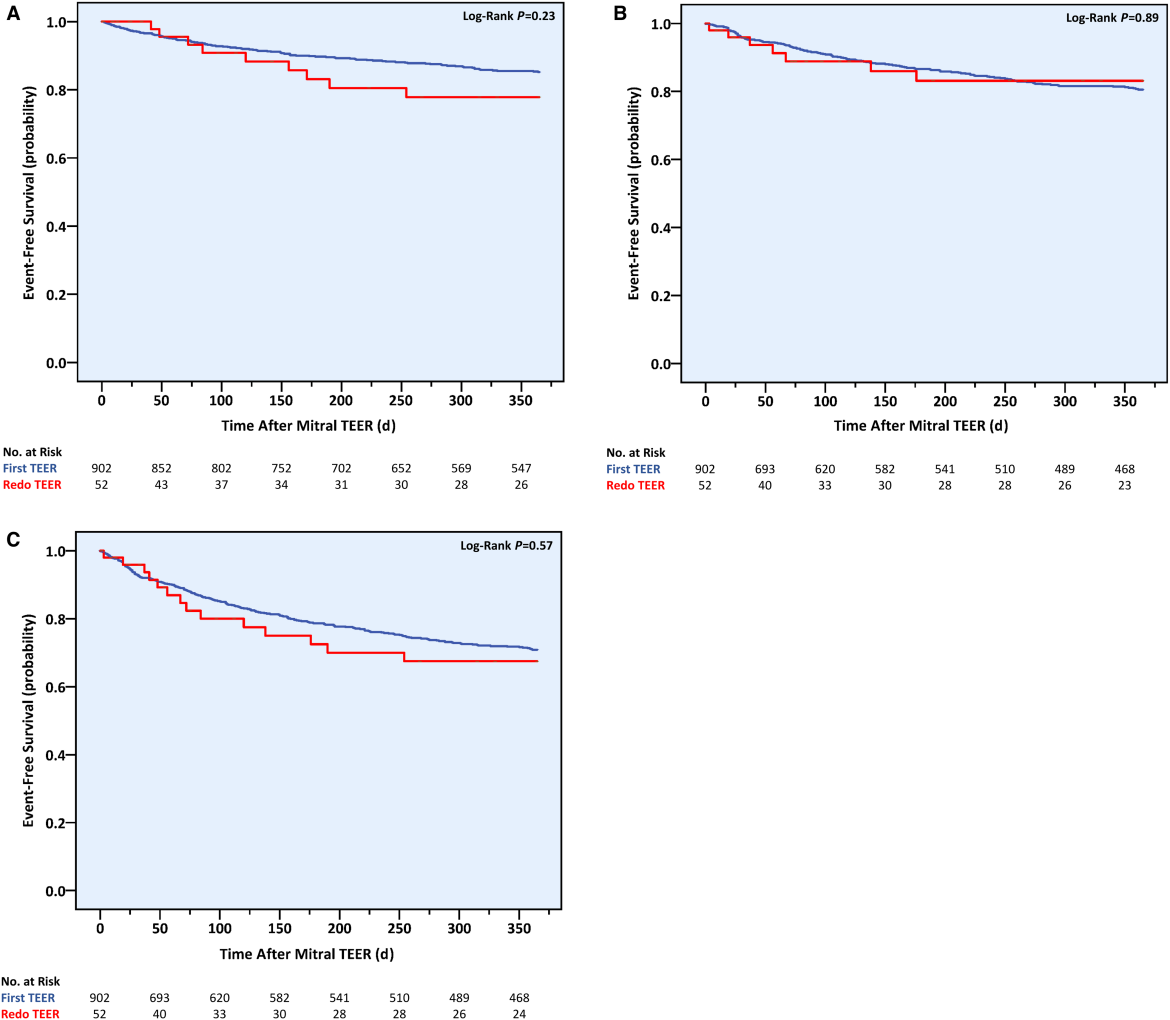
One‐year cumulative incidence of all‐cause death and heart failure hospitalization following first‐time and repeat, isolated mitral transcatheter edge‐to‐edge repair procedures. **A**, All‐cause death; **B**, heart failure hospitalizations; **C**, all‐cause death or heart failure hospitalizations. TEER indicates transcatheter edge‐to‐edge repair.

Compared with patients who underwent a first‐ever, isolated mitral TEER at CSMC without known reinterventions for 1 year (n=902), our redo cohort (n=52) was older and more functionally impaired, had a higher Society of Thoracic Surgeons score, was less likely to present with severe MR, and suffered more commonly from significant TR (Table S11). Intraprocedure, the redo group was treated with fewer, newer‐generation clips, which were more likely to be implanted in non‐A2P2 segments, mainly A3P3 (Table S12). Immediate and short‐term results were less optimal, as exemplified by lower rates of less than or equal to mild MR immediately after clip deployment and up to 1 month after the procedure (43.2% versus 63.7%; *P*=0.012), as well as less improvement in HF indices (such as Kansas City Cardiomyopathy Questionnaire 12 score and serum BNP level), a higher pulmonary arterial systolic pressure, and a higher rate of 1‐month significant TR (Tables S12 and S13). One‐year results were generally similar, including NYHA class, MR severity, transmitral mean pressure gradient, and echocardiographic measures of reverse remodeling and TR severity (Tables S12 and S13). These were accompanied by comparable 1‐year cumulative rates of all‐cause death (17.3% versus 12.1%; log‐rank *P*=0.23), HF hospitalizations (13.5% versus 15.0%; log‐rank *P*=0.89), and the composite of the 2 (26.9% versus 24.2%; log‐rank *P*=0.57; Figure 7).

By univariable analysis, redo TEER did not demonstrate an independent association with either of the clinical outcomes explored (all‐cause death: unadjusted HR, 1.51 [95% CI, 0.76–2.97]; *P*=0.24; HF hospitalizations: unadjusted HR, 0.95 [95% CI, 0.44–2.03]; *P*=0.89; primary outcome: unadjusted HR, 1.17 [95% CI, 0.68–2.01]; *P*=0.57). After incorporation of variables that differed between the first‐time and redo groups into a multivariable model, the primary outcome was again not associated with a repeat procedure but rather with NYHA class IV, significant TR, low tricuspid annular plane systolic excursion to pulmonary arterial systolic pressure ratio, and acute HF presentation (Table S14).

## Discussion

Our study evaluated the use of repeat mitral TEER for significant recurrent MR developing after a technically successful first intervention in a real‐world setting. Based on a large, single‐center registry in which redo procedures comprised close to 6% of all interventions, we observed the following:

1. All redo procedures were concluded by successful device deployment, without intraprocedural complications or conversion to surgery.

2. HF burden and MR were significantly reduced in almost all alive patients with available data up to 1‐year after the procedure, and complete freedom from severely symptomatic, significant MR was evident in almost three‐quarters.

3. A little more than 25% of patients experienced the primary outcome, a composite of all‐cause death, or HF hospitalization within the first postprocedural year.

4. Factors associated with an adverse clinical outcome were advanced HF presentation, functional MR cause, above‐moderate TR at baseline, urgent or early procedure (ie, within 1 month of the initial intervention), and non‐A2P2 (mainly A3P3) clipping.

5. Significant preprocedural TR emerged as an independent predictor of the primary outcome.

Currently, data on redo mitral TEER originate in retrospective or small studies that employed short‐term analyses. Studies exploring surgical approaches to TEER failure, on their part, have focused on less morbid and functionally impaired patients compared with redo mitral TEER cohorts, ours included, and mainly reported on death alone, which was mostly confined to the short‐term periprocedural period. In the largest report of redo mitral TEER before the present study, a strategy of an up‐front repeat procedure for a failed initial one (n=51) was associated with a trend toward reduced death compared with surgery (n=25) and medical therapy (n=71).^3^ However, baseline patient characteristics differed considerably between the treatment groups, and functional MR affected two‐thirds of the total cohort. Also, 7 redo procedures required surgical reintervention, and the follow‐up duration was relatively short (median, 162 days). Another study that specifically examined the association between MR cause and redo TEER outcomes showed a nonsignificantly improved survival following repeat procedure among patients with functional MR, but not primary MR, compared with patients referred to surgical intervention.^4^ Once again, major differences in preprocedural parameters were noted between the groups. An investigation that propensity‐matched 43 redo interventions with 43 conservatively treated cases after TEER failure reported a relative survival benefit in the redo TEER group.^5^ Here, patients displayed mainly (58.1%) functional MR and were followed for a short period (median, 9 months). Further, in most (58.1%) initial interventions, the discharge‐time MR was above moderate. Hence, by including the largest, most balanced cohort to date of redo mitral TEER cases, as well as by relying on 1 year of follow‐up, our study—although single‐arm—provides more robust data on procedural aspects and outcomes expected in this unique group of particularly morbid, high‐risk patients, the profile of whom was underrepresented in the surgical studies and substudies.

Despite the advanced HF status and elevated procedural risk encountered by our cohort, repeat mitral TEER proved universally feasible and safe and resulted in significant improvement in functional status and regurgitation severity compared with baseline among those who had available data. Moreover, its clinical effects matched those of the first procedure, even though health status deteriorated over time and was less optimal before the repeat intervention. Potential factors contributing to the encouraging outcomes of redo TEER at our institution included the use of newer‐generation devices and a lower number of devices per case, the absence of concomitant nonmitral interventions, and increased operator experience. While MR was less severe at the time of the repeat procedure, overall HF was more pronounced, suggesting that this apparently milder valvulopathy was underestimated, perhaps due to the presence of the prior clip(s).^23^, ^24^

Notably, amelioration of HF and MR following repeat mitral TEER was accompanied by somewhat less optimal structural results when compared with first‐time procedures. While achievement of up to moderate MR was comparable in the 2 groups, MR of up to a mild degree was observed in <40% of patients immediately after the redo clip deployment and in <60% of these cases at hospital discharge. Also, by 1 year, reverse cardiac remodeling by echo was not evident at all. In line with prior reports,^19^, ^25^, ^26^, ^27^ our findings likely mirrored the advanced disease state, which dictated a worse starting point for most redo TEER patients. In addition, the more complex interventional setting, signified by the abundance of urgent procedures, as well as the less ideal anatomic substrate for percutaneous edge‐to‐edge repair,^28^ as suggested in part by the rather high rate of non‐A2P2 targeting, may have all impaired the procedure's ability to fully address the recurrent valvular disorder. Finally, and specifically regarding the change in periprocedural LA pressure, the median of which matched a previous report,^29^ it could also be that some results were simply altered by the low number of observations.

Notwithstanding the structural effects of redo TEER, a clear survival benefit was shown in our study, with >80% of patients remaining alive after 1 year and almost three‐quarters being free of both death and HF hospitalizations. This survival rate exceeded those reported by previous smaller studies of patients undergoing redo mitral TEER^19^, ^25^, ^27^ and either matched^9^ or was only a few percentage points shy^8^ of those observed among surgical cohorts of failed TEER, the latter exhibiting considerably less functional impairment and fewer comorbidities than ours.

On a final note, no baseline mitral valve–related parameters, such as regurgitation severity and diastolic pressure gradients, nor the cause of MR recurrence, were independently associated with the primary outcome—although LV remodeling did impose a trend toward a higher event risk. Also, previously reported risk models (ie, Society of Thoracic Surgeons score and the MitraScore) were not discriminative of this composite end point. By contrast, HF indices, including NYHA class and serum BNP level, did correlate with adverse events, as did functional MR cause (which also was associated with the presence of LV remodeling), all being significantly more common in patients who experienced the primary outcome compared with patients who did not. Complementing the results of a previous study showing a considerably high (47%) 1‐year death rate among 17 patients with functional MR who underwent a repeat mitral TEER after a technically successful first intervention,^27^ these findings suggest that death and hospitalizations were mainly mediated by the underlying cardiomyopathy rather than the isolated valvular disease. In this regard, our study indicated above‐moderate TR at baseline as a marker of a less favorable outcome. A known risk factor for adverse postprocedural course in first‐time mitral TEER,^30^, ^31^, ^32^ significant TR in our cohort was associated with >3 times the risk for the primary outcome. Its presence may have pointed to irreversible cardiac damage that is beyond the therapeutic capacity of repeat mitral TEER or simply reflected a separate disorder that needs to be addressed by other means. Regardless of the mechanistic link, the prognostic importance of significant TR was further stressed by the observation that neither diuretic dose nor nonusage of common HF medications, all of which may affect TR, was associated with the primary outcome. In light of our observations, and pending future, prospective trials, MR cause, LV remodeling, and TR extent may dictate the patient selection process preceding redo mitral TEER.

### Limitations

First, our study is the product of a retrospective analysis of a small sample from a single, high‐volume center that did not use an external core laboratory. While this may impair generalizability of the results, we employed the largest cohort to date of redo mitral TEER procedures and relied on echocardiogram readings by blinded experts. Second, missing data, mainly regarding intraprocedural LA pressure as well as MR grade and functional status at 1 month and 1 year, could have interfered with the interpretation of the results and specifically limited our ability to evaluate the durability of MR repair. Nevertheless, the proportion of patients with missing data was comparable across the various subgroups; the availability of 1‐month data was not associated with the primary outcome; and baseline characteristics, deaths, and hospitalizations were reported in all patients. Third, the absence of significant MR recurrences at 1 year prevented us from drawing any conclusion pertaining to their possible predictors and consequently to considerations in favor of or against redo mitral TEER in this specific regard. Fourth, although representing a real‐world setting, and while driven by patients' features and tolerability, guideline‐directed therapy in our cohort was suboptimal in absolute terms. However, cardiac resynchronization therapy was not applied only in 2 patients with apparent indications: one with a class IIb indication and the other who refused an upgrade of his implantable cardioverter defibrillator. Fifth, the assessment of MR severity in preintervened valves could have been influenced by artifacts caused by existing clip(s). Moreover, we could not access echo images or reports preceding the first mitral TEER procedure or immediately following it in the 13 patients who underwent this first intervention outside of our institution. Finally, our results may not apply to non‐MitraClip systems, as those were not used in the study.

## Conclusion

Based on our single‐center experience, repeat mitral TEER for significant recurrent MR after a technically successful first procedure was feasible, safe, and clinically efficacious, especially among patients with nonfunctional MR without concomitant significant TR.

## Sources of Funding

The study was supported in part by the California Chapter of the American College of Cardiology through the Save a Heart Foundation.

## Disclosures

Dr Makkar received grant support from Edwards Lifesciences Corporation; is a consultant for Abbott Vascular, Cordis, and Medtronic; and holds equity in Entourage Medical. Dr Chakravarty is a consultant, proctor, and speaker for Edwards Lifesciences and Medtronic; is a consultant for Abbott Lifesciences; and is a consultant and speaker for Boston Scientific. The remaining authors have no disclosures to report.

## Supporting information

Tables S1–S14
