# Correction to: Repeat Mitral Transcatheter Edge‐to‐Edge Repair for Recurrent Significant Mitral Regurgitation

**DOI:** 10.1161/JAHA.122.027691

**Published:** 2023-06-01

In the article by Alon Shechter et al, “Repeat Mitral Transcatheter Edge‐to‐Edge Repair for Recurrent Significant Mitral Regurgitation,” which published online April 29, 2023 (*J Am Heart Assoc*. 2023;12:e028654. DOI: 10.1161/JAHA.122.028654) and was included in the May 2, 2023 issue of the journal, a correction was needed.

Due to a production error, the incorrect supplemental material file was published with this article. The correct supplement, containing Tables S1 through S14, has now been published with the article.

The publisher regrets the error.

The online version of the article has been updated and is available here: https://www.ahajournals.org/doi/10.1161/JAHA.122.028654.

