# Supplementary material for: Repeat Mitral Transcatheter Edge‐to‐Edge Repair for Recurrent Significant Mitral Regurgitation
Source: J Am Heart Assoc. 2023 Apr 29;12(9):e028654. doi: 10.1161/JAHA.122.028654 (PMC10227228; doi:10.1161/JAHA.122.028654)
Supplement: Supplementary file 1 — Tables S1–S14 [file JAH3-12-e028654-s001.pdf]

SUPPLEMENTAL MATERIAL

Table S1. Baseline Characteristics of the Study Population According to The Occurrence of the Primary Outcome.

	**Primary Outcome (N=14)**	**No Primary Outcome (N=38)**	**P-Value**
**Demographic Details**			
Age	82 (77-88)	81 (76-86)	0.79
Sex Male	6 (42.9)	23 (60.5)	0.26
**Medical Conditions**			
Non-Cardiovascular			
Body Mass Index (kg/m^2)	24.2 (22.1-26.1)	24.0 (21.9-25.9)	0.89
Diabetes Mellitus	3 (21.4)	11 (28.9)	0.73
Hypertension	13 (92.9)	33 (86.8)	1.00
Chronic Obstructive Pulmonary Disease	3 (21.4)	7 (18.4)	0.81
Anemia*	10 (71.4)	20 (52.6)	0.22
Stage ≥III Chronic Kidney Disease	13 (92.9)	25 (67.6)	0.08
Cardiovascular			
Previous MI, PCI, or CABG	7 (50.0)	11 (28.9)	0.20
Prior Stroke or TIA	1 (7.1)	6 (15.8)	0.66
Peripheral Arterial Disease	1 (7.1)	3 (7.9)	1.00
Atrial Fibrillation/Flutter	8 (57.1)	28 (73.7)	0.32
**Heart Failure Features**			
New York Heart Association Class			
II	0 (0.0)	3 (7.9)	0.56
III	1 (7.1)	11 (28.9)	0.14
IV	13 (92.9)	24 (63.2)	**0.043**
KCCQ12 Score	42.71 (31.25-56.43)	54.69 (24.09-68.88)	0.79
6-Minute Walk Test Distance (m)	122	243 (149-302)	0.60
Serum B-type Natriuretic Peptide (pg/mL)	1,059 (328-1,915)	442 (219-980)	**0.047**
**Procedural Risk**			
STS Score for Mitral Valve Repair	9.7 (3.6-16.2)	6.0 (3.4-12.0)	0.11
MitraScore	5 (3-5)	3 (2-4)	**0.040**
**Mitral Regurgitation Characteristics**			
Mitral Regurgitation Etiology			

Primary	3 (21.4)	20 (52.6)	**0.044**
Secondary / Functional	11 (78.6)	15 (39.5)	**0.012**
Mixed	0 (0.0)	3 (7.9)	0.56
Mitral Regurgitation Severity			
Moderate-Severe	2 (14.3)	12 (31.6)	0.14
Severe	12 (85.7)	26 (68.4)	0.81
Mitral Regurgitation PISA EROA (cm^2)	0.50 (0.32-0.50)	0.28 (0.20-0.50)	0.23
Mitral Regurgitation PISA RVol (mL)	45.8	30.6 (20.2-74.4)	0.75
Transmitral Mean Pressure Gradient (mmHg)	3 (2-4)	4 (3-5)	**0.040**
**Estimated Cause of Recurrent Mitral Regurgitation**			
Not Device-Related	3 (21.4)	13 (34.2)	0.51
Left Atrial Remodeling	1 (7.1)	4 (10.5)	
Left Ventricular Remodeling	4 (28.6)	4 (10.5)	
Prolapse/Flail Progression	6 (42.9)	17 (44.7)	
Device-Related	11 (78.6)	25 (65.8)	0.51
Grasping Loss	3 (21.4)	8 (21.1)	
Clip Migration	0 (0.0)	1 (2.6)	
Leaflet Detachment	0 (0.0)	4 (10.5)	
**Echocardiographic Indices**			
Left Heart			
Left Ventricular Ejection Fraction (%)	53 (19-61)	50 (31-63)	0.73
Left Ventricular End-Diastolic Diameter (cm)	5.5 (4.4-6.4)	5.0 (4.6-5.8)	0.38
Left Ventricular End-Systolic Diameter (cm)	4.1 (2.7-5.7)	3.6 (3.0-4.4)	0.44
Left Ventricular Mass Index, ASE Formula (gr/m^2)	122.91 (111.96-162.32)	110.99 (88.15-140.29)	0.37
Left Atrial Volume Index (cm^3/m^2)	56.0 (49.0-62.0)	53.5 (41.1-81.0)	0.98
Right Heart			
Right Ventricular Dysfunction	7 (58.3)	12 (32.3)	**0.013**
Right Ventricular Diameter (cm)	4.6 (3.8-5.4)	4.2 (3.7-4.8)	0.21
≥Moderate-Severe Tricuspid Regurgitation	10 (71.4)	9 (24.3)	**0.002**
Right Ventricular-Pulmonary Arterial Coupling			
TAPSE (mm)	16 (11-18)	18 (13-22)	0.13
PASP (mmHg)	40 (34-57)	47 (40-61)	0.30
TAPSE/PASP (mm/mmHg)	0.28 (0.27-0.29)	0.33 (0.26-0.48)	0.35
**Treatment**			

Medications			
Beta Blockers	9 (64.3)	25 (65.8)	0.92
RAS Inhibitors	6 (42.9)	20 (52.6)	0.53
MRAs	5 (35.7)	6 (15.8)	0.14
Loop Diuretics			
Frequency	14 (100.0)	30 (78.9)	0.09
Furosemide-equivalent dose (mg/day)	60 (40-120)	20 (20-70)	**0.015**
Devices			
Cardiac Implantable Electronic Device			
Total	9 (64.3)	10 (26.3)	**0.012**
Pacemaker	3 (21.4)	1 (2.6)	0.06
Implantable Cardioverter Defibrillator	3 (21.4)	2 (5.2)	0.11
Cardiac Resynchronization Therapy	0 (0.0)	0 (0.0)	NA
Cardiac Resynchronization Therapy Defibrillator	3 (21.4)	7 (18.4)	0.81
Hemodialysis	1 (7.1)	2 (5.3)	1.00
**Presentation and Preprocedural Course**			
Acute Decompensated Heart Failure	1 (7.1)	2 (5.3)	1.00
Cardiogenic Shock	1 (7.1)	0 (0.0)	0.27
IV Inotropic Support	1 (7.1)	2 (5.3)	1.00

Data are presented as number (percentage) or median (interquartile range). Figures in bold denote statistical significance.

* Anemia was defined as a blood hemoglobin level of <13mg/dL in men or <12mg/dL in women.

ASE = American Society of Echocardiography; CABG = coronary artery bypass grafting; EROA = effective regurgitant orifice area; IV = intravenous; KCCQ = Kansas City Cardiomyopathy Questionnaire; MI = myocardial infarction; MRAs = mineralocorticoid receptor antagonists; NA = not applicable; PASP = pulmonary arterial systolic pressure; PCI = percutaneous coronary intervention; PISA = proximal isovelocity surface area; RAS = renin-angiotensin system; RVol = regurgitant volume; STS = Society of Thoracic Surgeons; TAPSE = tricuspid annular plane systolic excursion; TIA = transient ischemic attack

Table S2. Procedural Details and Results According to the Occurrence of the Primary Outcome.

	Primary Outcome (N=14)	No Primary Outcome (N=38)	P-Value
**General Procedural Aspects**			
Time from First Procedure (days)			
Median (days)	164 (31-575)	438 (203-1,025)	**0.016**
≤One Month	3 (21.4)	0 (0.0)	**0.016**
≤One Year	9 (64.3)	14 (36.6)	0.08
Both Procedures at Cedars-Sinai Medical Center	11 (78.6)	31 (81.6)	0.81
Urgent Procedure	8 (57.1)	6 (15.8)	**0.005**
Total Duration (min)	124 (86-139)	100 (75-134)	0.18
Fluoroscopy Duration (min)	22 (16-32)	18 (14-28)	0.29
**Device Parameters**			
Clips Deployed			
1	7 (50.0)	28 (73.7)	0.18
≥2	7 (50.0)	10 (26.3)	0.18
Median	2 (1-2)	1 (1-2)	0.09
Device Generation			
1st	1 (7.1)	3 (7.9)	1.00
2nd	3 (21.4)	14 (36.8)	0.34
3rd	9 (64.3)	16 (42.1)	0.16
4th	1 (7.1)	5 (13.2)	0.55
Clip Site			
A1P1	0 (0.0)	2 (5.3)	1.00
A2P2	11 (78.6)	33 (86.8)	0.67
A3P3	5 (35.7)	3 (7.9)	**0.025**
**Immediate Postprocedural Effects**			
Echocardiography			
Mitral Regurgitation Severity ≤Mild			
After Clip Deployment	2 (14.3)	18 (47.4)	**0.030**
At Hospital Discharge	7 (50.0)	22 (59.5)	0.54
Mitral Regurgitation Severity ≤Moderate			

After Clip Deployment	14 (100.0)	38 (100.0)	1.00
At Hospital Discharge	12 (85.7)	37 (100.0)	0.07
Transmitral Mean Pressure Gradient (mmHg)	5 (3-5)	3 (2-5)	0.13
Pulmonary Venous Flow Pattern*			
Normalization on ≥1 Side	11 (78.6)	17 (53.1)	0.10
Improvement on ≥1 Side	12 (92.3)	15 (53.6)	**0.031**
Atrial Septal Defect			
Pre	10 (71.4)	17 (44.7)	0.09
Post	14 (100.0)	38 (100.0)	1.00
Right Heart Catheterization			
V wave (mmHg)			
Pre Clip Deployment	34 (22-38)	25 (17-40)	0.40
Post Clip Deployment	20 (17-32)	27 (19-36)	0.35
P-Value for Change	0.07	0.14	NA
Mean Left Atrial Pressure (mmHg)			
Pre Clip Deployment	21 (15-23)	17 (12-28)	0.42
Post Clip Deployment	17 (12-22)	20 (12-25)	0.44
P-Value for Change	0.08	0.56	NA
Mean Pulmary Arterial Pressure (mmHg)			
Pre Clip Deployment	29 (22-46)	34 (28-46)	0.45
Post Clip Deployment	21 (20-24)	30 (22-40)	0.15
P-Value for Change	0.11	0.48	NA
**Postprocedural Course**			
Intensive Care Unit Stay Duration (hours)	6.5±19.1	3.2±19.5	0.58
Hospitalization Length (days)	6 (1-15)	1 (1-3)	**0.002**
Discharge Home	13 (92.9)	38 (100.0)	0.27

Data are presented as number (percentage), median (interquartile range), or mean±standard deviation. Figures in bold denote statistical significance.

* Improvement and normalization of the pulmonary venous flow pattern were defined as a delta S/D velocities ratio of >1 and as a postprocedural S/D velocities ratio of ≥1, respectively.

Table S3. One-Month Heart Failure and Mitral Regurgitation Indices According to the Occurrence of the Primary Outcome.

	**Primary Outcome (N=14)**	**No Primary Outcome (N=38)**	**P-Value**
**Clinical**			
New York Heart Association Class			
I-II	2 (20.0)	22 (71.0)	**0.008**
Change from Baseline (classes)	-1±0.5	-1.4±0.8	0.10
P-Value vs Baseline	0.16	**<0.001**	NA
Improved from Baseline	9 (90.0)	27 (87.1)	1.00
KCCQ12 Score			
Median (points)	15.63	58.34 (46.09-99.22)	**0.021**
Change from Baseline (points)	-30.73	4.17 (-9.72-11.46)	0.25
P-Value vs Baseline	NA	0.60	NA
Improved from Baseline	0 (0.0)	4 (57.1)	0.29
Furosemide-Equivalent Dose			
Median (mg/day)	70 (0-80)	40 (0-40)	0.20
Change from Baseline (mg/day)	-30 (-70-38)	0 (-15-0)	0.20
P-Value vs Baseline	0.63	0.75	NA
Improved (reduced) from Baseline	7 (58.3)	9 (25.0)	0.07
**Laboratory**			
Serum B-type Natriuretic Peptide			
Median (pg/mL)	977 (265-3,242)	391 (173-723)	0.06
Change from Baseline (pg/mL)	88 (-336-539)	30 (-153-328)	0.56
P-Value vs Baseline	0.52	0.32	NA
Improved (reduced) from Baseline	3 (33.3)	6 (30.0)	1.00
**Echocardiographic**			
Mitral Regurgitation Severity ≤Mild; P-Value vs Baseline	2 (25.0); **0.003**	14 (48.3); **0.002**	0.42
Mitral Regurgitation Severity ≤Moderate; P-Value vs Baseline	6 (75.0); **<0.001**	27 (93.1); **<0.001**	0.20
Transmitral Mean Pressure Gradient			
Median (mmHg)	6 (4-7)	5 (3-7)	0.48
Median Change from Baseline (mmHg)	3 (0-5)	1 (-1-3)	0.14
Mean Change from Baseline (mmHg)	2.5±2.8	0.6±3.1	0.13

P-Value vs Baseline	**0.046**	0.46	NA
Left Ventricular Ejection Fraction			
Median (%)	40 (13-57)	56 (32-62)	0.34
Change from Baseline (%)	-4 (-6-2)	2 (-3-6)	0.17
P-Value vs Baseline	0.14	0.47	NA
Improved from Baseline	2 (28.6)	16 (57.1)	0.23
Left Ventricular End-Systolic Diameter			
Median (mm)	3.6 (2.9-4.6)	3.5 (2.7-4.3)	0.76
Change from Baseline (mm)	-0.1 (-0.3-0.5)	-0.2 (-0.5-0.3)	0.55
P-Value vs Baseline	0.69	0.52	NA
Improved (reduced) from Baseline	3 (50.0)	13 (61.9)	0.66
Left Atrial Volume Index			
Median (cm^3/m^2)	73.5 (45.1-94.5)	59.5 (41.2-76.8)	0.21
Change from Baseline (cm^3/m^2)	-12.7	-2.0 (-2.4-17.0)	0.50
P-Value vs Baseline	0.27	0.69	NA
Improved (reduced) from Baseline	1 (100.0)	7 (63.6)	0.46
Pulmonary Arterial Systolic Pressure			
Median (mmHg)	53 (48-61)	44 (33-54)	0.06
Change from Baseline (mmHg)	10 (6-24)	-2 (-12-11)	0.15
P-Value vs Baseline	0.07	0.89	NA
Improved (reduced) from Baseline	0 (0.0)	8 (50.0)	0.12
Tricuspid Regurgitation Severity ≥Moderate-Severe	4 (57.1)	10 (35.7)	0.40
P-Value vs Baseline	0.16	0.10	NA
**Combined**			
NYHA Class I-II and MR Severity ≤Mild; P-Value vs Baseline	0 (0.0); 0.16	9 (32.1); **<0.001**	0.08
NYHA Class I-II and MR Severity ≤Moderate; P-Value vs Baseline	1 (11.1); 0.17	19 (65.5); **<0.001**	**0.042**

Data are presented as number (percentage), median (interquartile range), or mean±standard deviation. Figures in bold denote statistical significance.

+ 1-year KCCQ12 results were documented in four patients only and therefore omitted from analysis for the no primary outcome group.

KCCQ = Kansas City Cardiomyopathy Questionnaire; MR = mitral regurgitation; NA = not applicable; NYHA = New York Heart Association

Table S4. Baseline Characteristics of the Study Population According to Mitral Regurgitation Etiology.

	Functional MR (N=26)	Non-Functional MR (N=26)	P-Value
**Demographic Details**			
Age	78 (74-82)	85 (80-89)	**0.004**
Sex Male	14 (53.8)	15 (57.7)	0.78
**Medical Conditions**			
Non-Cardiovascular			
Body Mass Ibdex (kg/m^2)	23.9 (22.2-26.0)	24.0 (21.7-25.9)	0.92
Diabetes Mellitus	9 (34.6)	5 (19.2)	0.21
Hypertension	24 (92.3)	22 (84.6)	0.67
Chronic Obstructive Pulmonary Disease	2 (7.7)	8 (30.8)	**0.035**
Anemia*	18 (69.2)	12 (46.2)	0.09
Stage ≥III Chronic Kidney Disease	21 (80.8)	17 (68.0)	0.30
Cardiovascular			
Previous MI, PCI, or CABG	15 (57.7)	3 (11.5)	**<0.001**
Prior Stroke or TIA	5 (19.2)	2 (7.7)	0.42
Peripheral Arterial Disease	3 (11.5)	1 (3.8)	0.61
Atrial Fibrillation/Flutter	16 (61.5)	20 (76.9)	0.23
**Heart Failure Features**			
New York Heart Association Class			0.63
II	1 (3.8)	2 (7.7)	
III	5 (19.2)	7 (26.9)	
IV	20 (76.9)	17 (65.4)	
KCCQ12 Score	57.47 (28.26-88.02)	46.53 (36.42-64.33)	0.44
6-Minute Walk Test Distance (m)	244 (114-305)	182 (149-287)	0.84
Serum B-type Natriuretic Peptide (pg/mL)	897 (304-2,089)	397 (196-810)	**0.030**
**Procedural Risk**			
STS Score for Mitral Valve Repair	8.3 (3.6-14.5)	5.9 (3.4-10.9)	0.39
MitraScore	4 (2-5)	3 (2-4)	**0.034**
**Mitral Regurgitation Characteristics**			
Mitral Regurgitation Severity			0.45

Moderate-Severe<br>Severe	6 (23.1)<br>20 (76.9)	8 (30.8)<br>18 (69.2)	
Mitral Regurgitation PISA EROA (cm^2)	0.26 (0.19-0.50)	0.47 (0.28-0.58)	0.21
Mitral Regurgitation PISA RVol (mL)	27.6 (19.9-57.6)	52.5 (30.6-61.2)	0.64
Transmitral Mean Pressure Gradient (mmHg)	4 (3-4)	4 (3-6)	0.41
**Estimated Cause of Recurrent Mitral Regurgitation**			
Not Device-Related<br>Left Atrial Remodeling<br>Left Ventricular Remodeling<br>Prolapse/Flail Progression	15 (57.7)<br>2 (7.7)<br>7 (26.9)<br>6 (23.1)	21 (80.8)<br>3 (11.5)<br>1 (3.8)<br>17 (65.4)	0.07<br><br>**0.021**
Device-Related<br>Grasping Loss<br>Clip Migration<br>Leaflet Detachment	11 (42.3)<br>8 (30.8)<br>1 (3.8)<br>2 (7.7)	5 (19.2)<br>3 (11.5)<br>0 (0.0)<br>2 (7.7)	0.07
**Echocardiographic Indices**			
Left Heart			
Left Ventricular Ejection Fraction (%)	27 (17-51)	56 (50-64)	**<0.001**
Left Ventricular End-Diastolic Diameter (cm)	5.6 (4.6-6.2)	4.9 (4.3-5.5)	**0.032**
Left Ventricular End-Systolic Diameter (cm)	4.4 (3.6-5.4)	3.2 (2.9-3.7)	**0.001**
Left Ventricular Mass Index, ASE Formula (gr/m^2)	135.25 (118.20-167.13)	104.31 (85.71-124.4)	**0.001**
Left Atrial Volume Index (cm^3/m^2)	56.0 (49.0-77.0)	50.5 (40.0-82.8)	0.86
Right Heart			
Right Ventricular Dysfunction	13 (56.5)	6 (24.0)	0.11
Right Ventricular Diameter (cm)	4.3 (3.9-5.1)	4.0 (3.6-4.6)	0.18
≥Moderate-Severe Tricuspid Regurgitation	12 (48.0)	7 (26.9)	0.12
Right Ventricular-Pulmonary Arterial Coupling			
TAPSE (mm)	16 (11-19)	18 (13-23)	0.27
PASP (mmHg)	41 (36-58)	49 (41-59)	0.27
TAPSE/PASP (mm/mmHg)	0.29 (0.26-0.40)	0.33 (0.28-0.47)	0.35
**Treatment**			
Medications			
Beta Blockers	20 (76.9)	14 (53.8)	0.08
RAS Inhibitors	13 (50.0)	13 (50.0)	1.00
MRAs	8 (30.8)	3 (11.5)	0.09

Loop Diuretics<br>Frequency<br>Furosemide-equivalent dose (mg/day)	 23 (88.5)<br>40 (25-120)	 21 (80.8)<br>20 (20-40)	 0.70<br>**0.015**
Devices			
Cardiac Implantable Electronic Device<br>Total<br>Pacemaker<br>Implantable Cardioverter Defibrillator<br>Cardiac Resynchronization Therapy<br>Cardiac Resynchronization Therapy Defibrillator	 16 (61.5)<br>2 (7.7)<br>4 (15.4)<br>0 (0.0)<br>10 (38.5)	 3 (11.5)<br>2 (7.7)<br>1 (3.8)<br>0 (0.0)<br>0 (0.0)	**<0.001**
Hemodialysis	2 (7.7)	1 (3.8)	0.55
**First Procedure Details**			
Time from First Procedure (days)<br>Median (days)<br>≤One Year	 395 (89-809)<br>13 (50.0)	 408 (156-1,003)<br>10 (38.5)	 0.60<br>0.40
First Procedure at Cedars-Sinai Medical Center	20 (76.9)	22 (84.6)	0.48
**Presentation and Preprocedural Course**			
Acute Decompensated Heart Failure	2 (7.7)	1 (3.8)	0.55
Cardiogenic Shock	0 (0.0)	1 (3.8)	0.31
IV Inotropic Support	1 (3.8)	2 (7.7)	0.55

Data are presented as number (percentage) or median (interquartile range). Figures in bold denote statistical significance.

* Anemia was defined as a blood hemoglobin level of <13mg/dL in men or <12mg/dL in women.

ASE = American Society of Echocardiography; CABG = coronary artery bypass grafting; EROA = effective regurgitant orifice area; IV = intravenous; KCCQ = Kansas City Cardiomyopathy Questionnaire; MI = myocardial infarction; MRAs = mineralocorticoid receptor antagonists; PASP = pulmonary arterial systolic pressure; PCI = percutaneous coronary intervention; PISA = proximal isovelocity surface area; RAS = renin-angiotensin system; RVol = regurgitant volume; STS = Society of Thoracic Surgeons; TAPSE = tricuspid annular plane systolic excursion; TIA = transient ischemic attack

Table S5. Procedural Details and Results According to Mitral Regurgitation Etiology.

	Functional MR<br>(N=26)	Non-Functional MR<br>(N=26)	P-Value
**General Procedural Aspects**			
Urgent Procedure	11 (42.3)	3 (11.5)	**0.012**
Total Duration (min)	103 (84-147)	100 (71-134)	0.28
Fluoroscopy Duration (min)	23 (16-34)	17 (13-26)	0.10
**Device Parameters**			
Clips Deployed			
1	16 (61.5)	19 (73.1)	0.38
≥2	10 (39.5)	7 (26.9)	0.38
Median	1 (1-2)	1 (1-2)	0.34
Device Generation			0.70
1st	1 (3.8)	3 (11.5)	
2nd	8 (30.8)	9 (34.6)	
3rd	14 (53.8)	11 (42.3)	
4th	3 (11.5)	3 (11.5)	
Clip Site			
A1P1	0 (0.0)	2 (7.7)	0.49
A2P2	23 (88.5)	21 (80.8)	0.70
A3P3	5 (19.2)	3 (11.5)	0.70
**Immediate Postprocedural Effects**			
Echocardiography			
Mitral Regurgitation Severity ≤Mild			
After Clip Deployment	7 (26.9)	13 (50.0)	0.09
At Hospital Discharge	18 (69.2)	11 (44.0)	0.07
Mitral Regurgitation Severity ≤Moderate			
After Clip Deployment	26 (100.0)	26 (100.0)	1.00
At Hospital Discharge	25 (96.2)	24 (96.0)	0.98
Transmitral Mean Pressure Gradient (mmHg)	4 (2-5)	4 (2-5)	0.78
Pulmonary Venous Flow Pattern*			
Normalization on ≥1 Side	14 (63.6)	14 (63.6)	0.71
Improvement on ≥1 Side	14 (73.7)	13 (59.1)	0.33

Atrial Septal Defect			
Pre	14 (53.8)	13 (50.0)	0.78
Post	26 (100.0)	26 (100.0)	1.00
Right Heart Catheterization			
V wave (mmHg)			
Pre Clip Deployment	35 (23-45)	24 (17-34)	0.07
Post Clip Deployment	29 (20-34)	21 (17-32)	0.15
P-Value for Change	0.10	0.17	NA
Mean Left Atrial Pressure (mmHg)			
Pre Clip Deployment	21 (17-29)	15 (13-20)	**0.033**
Post Clip Deployment	22 (15-26)	17 (11-21)	0.07
P-Value for Change	0.12	0.41	NA
Mean Pulmonary Arterial Pressure (mmHg)			
Pre Clip Deployment	34 (30-49)	32 (20-47)	0.42
Post Clip Deployment	33 (24-40)	25 (20-30)	0.28
P-Value for Change	0.46	0.35	NA
**Postprocedural Course**			
Intensive Care Unit Stay Duration (hours)	4.6±23.5	3.5±14.2	0.59
Hospitalization Length (days)	4 (1-8)	1 (1-2)	**0.010**
Discharge Home	25 (96.2)	26 (100.0)	1.00

Data are presented as number (percentage), median (interquartile range), or mean±standard deviation. Figures in bold denote statistical significance.

* Improvement and normalization of the pulmonary venous flow pattern were defined as a delta S/D velocities ratio of >1 and as a postprocedural S/D velocities ratio of ≥1, respectively.

MR = mitral regurgitation; PAP = pulmonary arterial pressure

Table S6. One-Month and One-Year Heart Failure and Mitral Regurgitation Indices According to Mitral Regurgitation Etiology.

	1-Month			1-Year		
	**Functional MR (N=26)**	**Non-Functional MR (N=26)**	**P-Value**	**Functional MR (N=26)**	**Non-Functional MR (N=26)**	**P-Value**
**Clinical**						
New York Heart Association Class						
I-II; P-Value vs Baseline	11 (57.9); **0.002**	13 (59.1); **0.001**	0.94	8 (80.0); **0.007**	10 (76.9); **0.002**	1.00
Change from Baseline (classes)	-1.3±0.7	-1.3±0.7	0.97	-1.5±0.9	-1.5±0.8	0.91
Improved (reduced) from Baseline	17 (89.5)	19 (86.4)	1.00	9 (90.0)	12 (92.3)	0.85
Furosemide-Equivalent Dose						
Median (mg/day); P-Value vs Baseline	40 (0-80); 0.42	40 (0-80); 0.92	0.36	120 (40-120); 0.14	40 (0-40); 0.79	**0.020**
Change from Baseline (mg/day)	0 (-40-0)	0 (-20-0)	0.43	5 (0-70)	0 (0-20)	0.27
Improved (reduced) from Baseline	10 (43.5)	6 (24.0)	0.15	1 (12.5)	1 (9.1)	1.00
**Laboratory**						
Serum B-type Natriuretic Peptide						
Median (pg/mL); P-Value vs Baseline	1,087 (642-2,787); 0.19	510 (227-1,137); 0.88	**<0.001**	1,063 (724-2,300); 0.46	524 (271-1,063); 0.06	**0.002**
Change from Baseline (pg/mL)	331 (-129-584)	23 (-189-48)	**0.037**	331 (-1,290-1,570)	54 (-8-85)	0.37
Improved (reduced) from Baseline	4 (26.7)	5 (35.7)	0.70	2 (33.3)	2 (28.6)	1.00
**Echocardiographic**						
Mitral Regurgitation Severity ≤Mild; P-Value vs Baseline	8 (44.4); **0.016**	8 (42.1); **0.025**	0.89	4 (50.0); 0.50	3 (25.0); 0.25	0.36
Mitral Regurgitation Severity ≤Moderate; P-Value vs Baseline	15 (83.3); **<0.001**	18 (94.7); **<0.001**	0.34	8 (100.0); **<0.001**	12 (100.0); **<0.001**	1.00
Transmitral Mean Pressure Gradient						

Median (mmHg); P-Value vs Baseline	5 (4-7); 0.07	5 (3-7); 0.51	0.46	3 (3-5); 0.26	4 (2-5); 0.40	0.76
Median Change from Baseline (mmHg)	1 (-1-1)	1 (-1-3)	0.27	-1 (-2-1)	-1 (-1-2)	0.43
Mean Change from Baseline (mmHg)	0.8±0.5	1.1±3.1	0.11	-0.8±1.5	0.2±2.4	0.23
Left Ventricular Ejection Fraction						
Median (%); P-Value vs Baseline	35 (17-43); 0.64	56 (32-61); 0.78	**<0.001**	23 (12-40); 0.85	60 (55-63); 0.48	**<0.001**
Change from Baseline (%)	0 (-4-5)	1 (-5-5)	0.76	4 (-5-13)	-3 (-10-13)	0.42
Improved from Baseline	8 (47.1)	10 (55.6)	0.62	4 (50.0)	5 (41.7)	1.00
Left Ventricular End-Systolic Diameter						
Median (mm); P-Value vs Baseline	4.4 (3.1-5.6); 0.43	3.5 (2.7-4.4); 0.10	**0.001**	5.9 (4.5-8.2); 0.67	3.2 (2.6-4.0); 0.78	**0.001**
Change from Baseline (mm)	0.0 (-0.2-0.7)	-0.1 (-0.4-0.3)	0.11	0.0 (-0.6-0.4)	0.0 (-0.1-0.5)	1.00
Improved (reduced) from Baseline	6 (46.2)	10 (71.4)	0.18	3 (50.0)	3 (33.3)	0.62
Left Atrial Volume Index						
Median (cm^3/m^2); P-Value vs Baseline	63.0 (43.3-84.3);0.61	61.9 (41.9-80.0); 0.58	0.38	49.5 (36.2-56.7); 0.55	54.0 (39.9-76.1); 0.59	1.00
Change from Baseline (cm^3/m^2)	-2.0 (-13.4-12.9)	-2.0 (-10.1-15.8)	0.88	-56.0 (-79.0-[-50.0])	-55.5 (-81.0-[-41.5])	0.89
Improved (reduced) from Baseline	4 (80.0)	4 (57.1)	0.58	13 (100.0)	14 (93.3)	0.34
Pulmonary Arterial Systolic Pressure						
Median (mmHg); P-Value vs Baseline	50 (45-56); 0.07	48 (37-56); 0.44	0.08	54 (37-59); 0.47	44 (30-52); 0.58	0.50
Change from Baseline (mmHg)	9 (1-24)	6 (-10-12)	**0.038**	-6 (-18-5)	1 (-21-20)	0.52
Improved (reduced) from Baseline	2 (22.2)	6 (54.5)	.020	4 (80.0)	3 (50.0)	0.55
TR Severity ≥Moderate-Severe; P-Value vs Baseline	7 (41.2); 0.66	7 (38.9); 0.08	0.89	3 (37.5); 0.25	3 (25.0); 0.87	0.64
**Combined**						
NYHA Class I-II and MR Severity ≤Mild; P-Value vs Baseline	5 (27.8); **0.001**	4 (21.1); **0.002**	0.71	3 (37.5); 0.14	2 (16.7); 0.16	0.35
NYHA Class I-II and MR Severity ≤Moderate; P-Value vs Baseline	9 (50.0); **<0.001**	11 (57.9); **<0.001**	0.76	5 (71.4); 0.10	9 (75.0); 0.10	1.00

Data are presented as number (percentage), median (interquartile range), or mean±standard deviation. Figures in bold denote statistical significance.

MR = mitral regurgitation; NYHA = New York Heart Association; TR = tricuspid regurgitation

Table S7. Clinical Outcomes According to Mitral Regurgitation Etiology.

	1-Month			1-Year			1-Year Event-Free Survival		
	Functional MR (N=26)	Non-Functional MR (N=26)	P-Value	Functional MR (N=26)	Non-Functional MR (N=26)	P-Value	Functional MR (N=26)	Non-Functional MR (N=26)	P-Value
**Primary Outcome**									
All-Cause Mortality or Heart Failure Hospitalizations	1 (3.8)	1 (3.8)	1.00	11 (42.3)	3 (11.5)	**0.012**	227.7±30.6	334.5±17.5	**0.016**
**Secondary Outcomes**									
All-Cause Mortality	0 (0.0)	0 (0.0)	NA	7 (26.9)	2 (7.7)	0.14	279.6±27.1	348.9±11.2	0.06
Heart Failure Hospitalizations	1 (3.8)	1 (3.8)	1.00	5 (19.2)	2 (7.7)	0.42	293.8±28.0	340.4±16.9	0.21
Mitral Re-Intervention	0 (0.0)	0 (0.0)	NA	2 (7.7)	1 (3.8)	0.55	337.1±18.9	349.9±14.7	0.59
MI / Stroke / TIA	0 (0.0)	1 (3.8)	1.00	0 (0.0)	2 (7.7)	0.49	NA	110.7±58.5	NA
Major Bleeding	1 (3.8)	0 (0.0)	1.00	2 (7.7)	0 (0.0)	0.49	130.0±109.0	NA	NA

Data are presented as number (percentage) or mean±standard deviation. Figures in bold denote statistical significance.

MI = myocardial infarction; MR = mitral regurgitation; TIA = transient ischemic attack

Table S8. Baseline Characteristics of Patients that Underwent Both First and Redo Procedures at Cedars-Sinai.

	**First Procedure (N=39)**	**Redo Procedure (N=39)**	**P-Value**
**Demographic Details**			
Age	80 (75-86)	81 (76-88)	0.43
Sex Male	20 (51.3)	20 (51.3)	1.00
**Medical Conditions**			
Non-Cardiovascular			
Body Mass Index (kg/m^2)	23.8 (22.2-25.7)	23.8 (21.8-25.9)	0.68
Diabetes Mellitus	8 (20.5)	9 (23.1)	0.78
Hypertension	35 (89.7)	34 (87.2)	0.72
Chronic Obstructive Pulmonary Disease	8 (20.5)	10 (25.6)	0.59
Anemia*	17 (43.6)	20 (51.3)	0.50
Stage ≥III Chronic Kidney Disease	29 (74.4)	30 (78.9)	0.63
Cardiovascular			
Previous MI, PCI, or CABG	12 (30.8)	12 (30.8)	1.00
Prior Stroke or TIA	3 (7.7)	4 (10.3)	1.00
Peripheral Arterial Disease	2 (5.3)	4 (10.3)	0.41
Atrial Fibrillation/Flutter	23 (59.0)	28 (71.8)	0.23
**Heart Failure Features**			
New York Heart Association Class			
II	5 (12.8)	3 (7.7)	0.71
III	18 (46.2)	6 (15.4)	**0.003**
IV	16 (41.0)	30 (76.9)	**0.001**
KCCQ12 Score	48.96 (31.25-63.54)	56.25 (27.87-67.97)	0.53
6-Minute Walk Test Distance (m)	259 (206-339)	244 (149-302)	0.28
Serum B-type Natriuretic Peptide (pg/mL)	384 (216-578)	730 (310-1,453)	0.07
**Procedural Risk**			
STS Score for Mitral Valve Repair	6.0 (3.0-8.0)	7.2 (3.6-13.7)	0.14
MitraScore	3 (2-4)	4 (3-5)	0.08
**Mitral Regurgitation Characteristics**			
Mitral Regurgitation Etiology			1.00

Primary<br>Secondary/Functional<br>Mixed	22 (56.4)<br>17 (43.6)<br>0 (0.0)	22 (56.4)<br>17 (43.6)<br>0 (0.0)	
Mitral Regurgitation Severity<br>Moderate-Severe<br>Severe	 1 (2.6)<br>38 (97.4)	 10 (25.6)<br>29 (74.4)	**0.018**
Mitral Regurgitation PISA EROA (cm^2)	0.39 (0.30-0.51)	0.26 (0.18-0.50)	0.13
Mitral Regurgitation PISA RVol (mL)	59.0 (36.0-79.0)	30.6 (20.2-45.8)	**0.035**
Transmitral Mean Pressure Gradient (mmHg)	2 (2-3)	4 (3-5)	**0.001**
**Estimated Cause of Recurrent Mitral Regurgitation**			
Not Device-Related<br>Left Atrial Remodeling<br>Left Ventricular Remodeling<br>Prolapse/Flail Progression	NA	26 (66.7)<br>3 (7.7)<br>7 (17.9)<br>16 (41.0)	NA
Device-Related<br>Grasping Loss<br>Clip Migration<br>Leaflet Detachment	NA	13 (33.3)<br>9 (23.1)<br>1 (2.6)<br>3 (7.7)	NA
**Echocardiographic Indices**			
Left Heart			
Left Ventricular Ejection Fraction (%)	59 (29-66)	50 (28-56)	**0.016**
Left Ventricular End-Diastolic Diameter (cm)	5.4 (4.8-6.3)	5.2 (4.6-5.9)	0.17
Left Ventricular End-Systolic Diameter (cm)	3.5 (3.0-5.2)	3.8 (3.0-4.6)	0.95
Left Ventricular Mass Index, ASE Formula (gr/m^2)	130.84 (99.07-159.24)	116.72 (93.32-140.84)	0.36
Left Atrial Volume Index (cm^3/m^2)	59.0 (42.0-71.0)	52.0 (40.0-77.0)	0.79
Right Heart			
Right Ventricular Dysfunction	10 (28.6)	16 (43.2)	0.20
Right Ventricular Diameter (cm)	4.0 (3.6-4.4)	4.2 (3.7-4.8)	0.39
≥Moderate-Severe Tricuspid Regurgitation	12 (30.8)	16 (42.1)	0.30
Right Ventricular-Pulmonary Arterial Coupling			
TAPSE (mm)	17 (14-20)	17 (11-21)	0.54
PASP (mmHg)	44 (33-56)	46 (40-59)	0.60
TAPSE/PASP (mm/mmHg)	0.40 (0.30-0.50)	0.29 (0.26-0.41)	**0.023**
**Treatment**			

Medications			
Beta Blockers	26 (66.7)	28 (71.8)	0.63
RAS Inhibitors	24 (61.5)	19 (48.7)	0.26
MRAs	5 (12.8)	8 (20.5)	0.36
Loop Diuretics			
Frequency	29 (74.4)	34 (87.2)	0.15
Furosemide-equivalent dose (mg/day)	20 (10-40)	40 (20-80)	0.07
Devices			
Cardiac Implantable Electronic Device			0.60
Total	11 (28.2)	15 (38.5)	
Pacemaker	3 (7.6)	4 (10.3)	
Implantable Cardioverter Defibrillator	1 (2.6)	3 (7.6)	
Cardiac Resynchronization Therapy	1 (2.6)	0 (0.0)	
Cardiac Resynchronization Therapy Defibrillator	6 (15.4)	8 (20.6)	
Hemodialysis	2 (5.3)	2 (5.3)	1.00
**Presentation and Preprocedural Course**			
Acute Decompensated Heart Failure	2 (5.1)	2 (5.1)	1.00
Cardiogenic Shock	1 (2.6)	1 (2.6)	1.00
IV Inotropic Support	1 (2.6)	3 (7.7)	0.62

Data are presented as number (percentage) or median (interquartile range). Figures in bold denote statistical significance.

* Anemia was defined as a blood hemoglobin level of <13mg/dL in men or <12mg/dL in women.

CABG = coronary artery bypass grafting; EROA = effective regurgitant orifice area; IV = intravenous; KCCQ = Kansas City Cardiomyopathy Questionnaire; MI = myocardial infarction; MRAs = mineralocorticoid receptor antagonists; NA = not applicable; PASP = pulmonary arterial systolic pressure; PCI = percutaneous coronary intervention; PISA = proximal isovelocity surface area; RAS = renin-angiotensin system; RVol = regurgitant volume; STS = Society of Thoracic Surgeons; TAPSE = tricuspid annular plane systolic excursion; TIA = transient ischemic attack

Table S9. Procedural Details and Results of First and Redo Procedures Performed at Cedars-Sinai on the Same Patients.

	First Procedure (N=39)	Redo Procedure (N=39)	P-Value
**General Procedural Aspects**			
Time from First Procedure (days)	NA		NA
Median (days)		373 (144-805)	
≤One Year		19 (48.7)	
Urgent Procedure	6 (15.4)	12 (30.8)	0.11
Total Duration (min)	116 (90-146)	110 (84-145)	0.94
Fluoroscopy Duration (min)	20 (14-27)	19 (14-34)	0.53
Concomitant Interventions (ASD Closure, TAVR, Tricuspid TEER)	5 (12.8)	0 (0.0)	0.06
Conversion to Surgery	0 (0.0)	0 (0.0)	NA
Intraprocedural Complications	0 (0.0)	0 (0.0)	NA
**Device Parameters**			
Clips Deployed			
1	20 (51.3)	27 (69.2)	0.11
≥2	19 (48.7)	12 (30.8)	0.11
Median	1 (1-2)	1 (1-2)	0.08
Device Generation			**0.002**
1st	15 (38.5)	3 (7.7)	**0.001**
2nd	12 (30.8)	10 (25.6)	0.62
3rd	11 (28.2)	20 (51.3)	**0.037**
4th	1 (2.6)	6 (15.4)	0.11
Intervention Site			
A1P1	0 (0.0)	2 (5.1)	0.49
A2P2	38 (97.4)	32 (82.1)	0.06
A3P3	3 (7.7)	7 (17.9)	0.18
**Immediate Postprocedural Effects**			
Echocardiography			
Mitral Regurgitation Severity ≤Mild			
After Clip Deployment	27 (69.2)	14 (35.9)	**0.003**
At Hospital Discharge	30 (76.9)	20 (52.6)	**0.016**

Mitral Regurgitation Severity ≤Moderate			
After Clip Deployment	38 (97.4)	39 (100.0)	1.00
At Hospital Discharge	36 (92.3)	36 (94.7)	1.00
Transmitral Mean Pressure Gradient (mmHg)	3 (2-4)	3 (2-5)	0.15
Pulmonary Venous Flow Pattern*			
Normalization on ≥1 Side	20 (54.1)	21 (60.0)	0.61
Improvement on ≥1 Side	26 (76.5)	20 (64.5)	0.29
Right Heart Catheterization			
V wave (mmHg)			
Pre	30 (20-40)	28 (18-38)	0.83
Post	21 (13-26)	26 (18-34)	**0.048**
P-Value for Change	**0.001**	**0.045**	NA
Mean Left Atrial Pressure (mmHg)			
Pre	18 (13-23)	17 (14-27)	0.80
Post	14 (10-19)	19 (13-24)	**0.016**
P-Value for Change	**0.003**	0.19	NA
Mean Pulmonary Arterial Pressure (mmHg)			
Pre	27 (23-30)	34 (23-47)	0.04
Post	25 (22-30)	28 (21-38)	0.91
P-Value for Change	0.51	0.26	NA
**Postprocedural Course**			
Intensive Care Unit Stay Duration (hours)	1.8±6.2	2.3±11.6	0.64
Hospitalization Length (days)	1 (1-2)	1 (1-5)	0.29
Discharge Home	39 (100.0)	39 (100.0)	NA

Data are presented as number (percentage), median (interquartile range), or mean±standard deviation. Figures in bold denote statistical significance.

* Improvement and normalization of the pulmonary venous flow pattern were defined as a delta S/D velocities ratio of >1 and as a postprocedural S/D velocities ratio of ≥1, respectively.

ASD = atrial septal defect; NA = not applicable; PAP = pulmonary arterial pressure; TAVR = transcatheter aortic valve replacement; TEER = transcatheter edge-to-edge repair

Table S10. One-Month Heart Failure and Mitral Regurgitation Indices Following First and Redo Procedures Performed at Cedars-Sinai on the Same Patients More Than a Month Apart.

	**First Procedure (N=37)**	**Redo Procedure (N=37)**	**P-Value**
**Clinical**			
New York Heart Association Class			
I-II	22 (64.7)	16 (57.1)	0.54
Change from Baseline (classes)	-1.1±0.8	-1.3±0.8	0.25
P-Value vs Baseline	**<0.001**	**<0.001**	NA
Improved from Baseline	30 (88.2)	24 (85.7)	1.00
Furosemide-Equivalent Dose			
Median (mg/day)	20 (0-70)	40 (20-80)	0.17
Change from Baseline (mg/day)	0 (-10-0)	0 (-15-0)	0.62
P-Value vs Baseline	0.80	0.62	NA
Improved (reduced) from Baseline	10 (27.8)	9 (25.0)	0.80
**Laboratory**			
Serum B-type Natriuretic Peptide			
Median (pg/mL)	411 (267-1,210)	411 (220-1,188)	0.77
Change from Baseline (pg/mL)	92 (-81-330)	24 (-191-324)	0.46
P-Value vs Baseline	0.06	0.75	NA
Improved (reduced) from Baseline	8 (36.4)	8 (40.0)	1.00
**Echocardiographic**			
Mitral Regurgitation Severity ≤Mild			
After Clip Deployment	26 (70.3)	14 (37.8)	**0.005**
At 1-Month	12 (37.5)	10 (40.0)	0.85
Mitral Regurgitation Severity ≤Moderate			
After Clip Deployment	36 (97.3)	37 (100.0)	1.00
At 1-Month	27 (84.4)	23 (92.0)	0.45
Transmitral Mean Pressure Gradient			
Median (mmHg)	4 (3-5)	5 (3-7)	0.18
Median Change from Baseline (mmHg)	1 (0-2)	(-1-3)	0.40

Mean Change from Baseline (mmHg)	1.5±2.3	0.8±3.4	0.37
P-Value vs Baseline	**<0.001**	0.40	NA
Left Ventricular Ejection Fraction			
Median (%)	56 (29-61)	56 (32-60)	0.92
Change from Baseline (%)	-4 (-10-1)	2 (0-6)	**0.005**
P-Value vs Baseline	**0.005**	0.21	NA
Improved from Baseline	8 (25.0)	16 (69.6)	**0.001**
Left Ventricular End-Ssytolic Diameter			
Median (mm)	3.7 (2.9-4.9)	3.2 (2.7-4.3)	0.25
Change from Baseline (mm)	-0.1 (-0.5-0.4)	-0.2 (-0.7-[-0.1])	0.10
P-Value vs Baseline	0.78	**0.025**	NA
Improved (reduced) from Baseline	16 (50.0)	14 (82.4)	**0.027**
Left Atrial Volume Index			
Median (cm^3/m^2)	49.8 (37.5-79.8)	63.0 (47.0-80.0)	0.23
Change from Baseline (cm^3/m^2)	0.0 (-22.5-17.4)	-1.5 (-5.0-18.0)	0.74
P-Value vs Baseline	0.63	0.59	NA
Improved (reduced) from Baseline	14 (48.3)	6 (60.0)	0.72
Pulmonary Arterial Systolic Pressure			
Median (mmHg)	44 (36-49)	47 (35-57)	0.22
Change from Baseline (mmHg)	-3 (-10-4)	5 (-11-18)	0.29
P-Value vs Baseline	0.21	0.58	NA
Improved (reduced) from Baseline	17 (58.6)	4 (36.4)	0.21
Tricuspid Regurgitation Severity ≥Moderate-Severe	7 (21.9)	11 (45.8)	0.06
P-Value vs Baseline	0.69	0.45	NA
**Combined**			
NYHA Class I-II and MR Severity ≤Mild; P-Value vs Baseline	10 (30.3); **<0.001**	4 (15.4); **0.024**	0.21
NYHA Class I-II and MR Severity ≤Moderate; P-Value vs Baseline	19 (57.6); **<0.001**	13 (50.0); **<0.001**	0.56

Data are presented as number (percentage), median (interquartile range), or mean±standard deviation. Figures in bold denote statistical significance.

MR = mitral regurgitation; NA = not applicable; NYHA = New York Heart Association

Table S11. Baseline Characteristics of Patients that Underwent Either the First or the Redo Procedure at Cedars-Sinai.

	First Procedure (N=902)	Redo Procedure (N=52)	P-Value
**Demographic Details**			
Age	79 (70-86)	81 (76-87)	**0.024**
Sex Male	541 (60.0)	29 (55.8)	0.55
**Medical Conditions**			
Non-Cardiovascular			
Body Mass Index (kg/m^2)	24.24 (21.63-28.00)	24.0 (22.0-25.9)	0.41
Diabetes Mellitus	239 (26.6)	14 (26.9)	0.96
Hypertension	749 (83.1)	46 (88.5)	0.32
Chronic Obstructive Pulmonary Disease	119 (13.2)	10 (19.2)	0.22
Anemia*	567 (62.9)	30 (57.7)	0.45
Stage ≥III Chronic Kidney Disease	646 (73.4)	38 (74.5)	0.86
Cardiovascular			
Previous MI, PCI, or CABG	394 (43.7)	18 (34.6)	0.20
Prior Stroke or TIA	124 (13.7)	7 (13.5)	0.95
Peripheral Arterial Disease	70 (7.8)	4 (7.7)	1.00
Atrial Fibrillation/Flutter	475 (52.7)	36 (69.2)	**0.020**
**Heart Failure Features**			
New York Heart Association Class			0.06
II	56 (6.2)	3 (5.8)	1.00
III	371 (41.2)	12 (23.1)	**0.010**
IV	472 (52.4)	37 (71.2)	**0.008**
KCCQ12 Score	38.02 (18.23-61.46)	53.22 (24.01-66.32)	0.13
6-Minute Walk Test Distance (m)	244 (122-335)	214 (120-288)	0.56
Serum B-type Natriuretic Peptide (pg/mL)	507 (237-1,243)	480 (223-1,141)	0.89
**Procedural Risk**			
STS Score for Mitral Valve Repair	5.6 (3.0-8.9)	6.6 (3.7-13.3)	**0.039**
MitraScore	3 (2-4)	4 (2-5)	0.34
**Mitral Regurgitation Characteristics**			
Mitral Regurgitation Etiology			0.97

Primary<br>Secondary/Functional<br>Mixed	386 (42.8)<br>466 (51.7)<br>50 (5.5)	23 (44.2)<br>26 (50.0)<br>3 (5.8)	0.84<br>0.82<br>0.76
Mitral Regurgitation Severity<br>Moderate-Severe<br>Severe	 179 (19.8)<br>722 (80.0)	 14 (26.9)<br>38 (73.1)	0.12<br>**0.046**<br>**0.029**
Mitral Regurgitation PISA EROA (cm^2)	0.35 (0.26-0.49)	0.37 (0.20-0.50)	0.68
Mitral Regurgitation PISA RVol (mL)	50.8 (36.8-67.6)	31.4 (20.9-67.2)	0.08
Transmitral Mean Pressure Gradient (mmHg)	3 (2-4)	4 (3-5)	**<0.001**
**Echocardiographic Indices**			
Left Heart			
Left Ventricular Ejection Fraction (%)	51 (30-63)	50 (26-62)	0.40
Left Ventricular End-Diastolic Diameter (cm)	5.3 (4.7-6.1)	5.0 (4.6-5.9)	0.16
Left Ventricular End-Systolic Diameter (cm)	3.9 (3.1-5.0)	3.6 (3.0-4.7)	0.28
Left Ventricular Mass Index, ASE Formula (gr/m^2)	125.9 (101.5-152.8)	119.7 (93.3-140.8)	0.30
Left Atrial Volume Index (cm^3/m^2)	57.0 (43.2-73.1)	55.0 (44.2-81.0)	0.90
Right Heart			
Right Ventricular Dysfunction	329 (42.2)	19 (44.2)	0.64
Right Ventricular Diameter (cm)	4.1 (3.5-4.6)	4.2 (3.8-4.8)	0.29
≥Moderate-Severe Tricuspid Regurgitation	196 (21.8)	19 (37.3)	**0.010**
Right Ventricular-Pulmonary Arterial Coupling			
TAPSE (mm)	17 (14-20)	17 (11-21)	0.44
PASP (mmHg)	45 (34-57)	45 (39-59)	0.63
TAPSE/PASP (mm/mmHg)	0.37 (0.27-0.56)	0.31 (0.27-0.43)	0.10
**Treatment**			
Medications			
Beta Blockers	624 (69.2)	34 (65.4)	0.57
RAS Inhibitors	446 (49.4)	26 (50.0)	0.94
MRAs	185 (20.5)	11 (21.2)	0.91
Loop Diuretics<br>Frequency<br>Furosemide-equivalent dose (mg/day)	 672 (74.5)<br>40 (0-60)	 44 (84.6)<br>40 (20-80)	 0.10<br>0.18
Devices			
Cardiac Implantable Electronic Device			0.20

Total	298 (33.0)	19 (36.5)	0.60
Pacemaker	103 (11.4)	4 (7.7)	0.41
Implantable Cardioverter Defibrillator	61 (6.8)	5 (9.6)	0.40
Cardiac Resynchronization Therapy	29 (3.2)	0 (0.0)	0.40
Cardiac Resynchronization Therapy Defibrillator	105 (11.6)	10 (19.2)	0.10
Hemodialysis	75 (8.3)	3 (5.8)	0.79
**Presentation and Preprocedural Course**			
Acute Decompensated Heart Failure	143 (15.9)	3 (5.8)	0.05
Cardiogenic Shock	25 (2.8)	1 (1.9)	0.72
IV Inotropic Support	47 (5.2)	3 (5.8)	0.75
Mechanical Assist Device	16 (1.8)	0 (0.0)	0.82

Data are presented as number (percentage) or median (interquartile range). Figures in bold denote statistical significance.

* Anemia was defined as a blood hemoglobin level of <13mg/dL in men or <12mg/dL in women.

CABG = coronary artery bypass grafting; EROA = effective regurgitant orifice area; IV = intravenous; KCCQ = Kansas City Cardiomyopathy Questionnaire; MI = myocardial infarction; MRAs = mineralocorticoid receptor antagonists; PASP = pulmonary arterial systolic pressure; PCI = percutaneous coronary intervention; PISA = proximal isovelocity surface area; RAS = renin-angiotensin system; RVol = regurgitant volume; STS = Society of Thoracic Surgeons; TAPSE = tricuspid annular plane systolic excursion; TIA = transient ischemic attack

Table S12. Procedural Details and Results of First and Redo Procedures Performed at Cedars-Sinai on Different Patients.

	First Procedure (N=902)	Redo Procedure (N=52)	P-Value
**General Procedural Aspects**			
Urgent Procedure	166 (18.4)	14 (26.9)	0.13
Total Duration (min)	108 (86-136)	111 (80-135)	0.32
Fluoroscopy Duration (min)	19 (14-26)	18 (15-29)	0.45
Conversion to Surgery	1 (0.1)	0 (0.0)	1.00
Intraprocedural Complications	4 (4.4)	0 (0.0)	0.73
**Device Parameters**			
Clips Deployed			
0 (aborted / not deployed)	18 (2.0)	0 (0.0)	0.62
1	388 (43.0)	35 (67.3)	**0.001**
≥2	496 (55.0)	17 (32.7)	**0.002**
Median	2 (1-2)	1 (1-2)	**0.001**
Device Generation			
1st	286 (31.7)	4 (7.7)	**<0.001**
2nd	293 (32.5)	17 (32.7)	0.98
3rd	218 (24.2)	25 (48.1)	**<0.001**
4th	105 (11.6)	6 (11.5)	0.98
Intervention Site			
A1P1	27 (3.0)	2 (3.8)	0.67
A2P2	863 (95.7)	44 (84.6)	**0.003**
A3P3	51 (5.7)	8 (15.4)	**0.012**
**Immediate Postprocedural Effects**			
Echocardiography			
Mitral Regurgitation Severity ≤Mild			
After Clip Deployment	652 (72.3)	20 (38.5)	**<0.001**
At Hospital Discharge	700 (79.5)	29 (56.9)	**<0.001**
Mitral Regurgitation Severity ≤Moderate			
After Clip Deployment	870 (97.1)	52 (100.0)	0.39
At Hospital Discharge	861 (97.7)	49 (96.1)	0.97

Transmitral Mean Pressure Gradient (mmHg)	3 (2-4)	4 (2-5)	0.05
Pulmonary Venous Flow Pattern*<br>Normalization on ≥1 Side<br>Improvement on ≥1 Side	 516 (65.1)<br>590 (78.4)	 28 (60.9)<br>27 (65.9)	 0.56<br>0.06
Right Heart Catheterization			
V wave (mmHg)<br>Pre<br>Post<br>P-Value for Change	 30 (20-44)<br>20 (15-27)<br>**<0.001**	 28 (18-39)<br>25 (19-33)<br>**0.027**	 0.35<br>**0.011**<br>NA
Mean Left Atrial Pressure (mmHg)<br>Pre<br>Post<br>P-Value for Change	 19 (13-26)<br>15 (11-20)<br>**<0.001**	 18 (14-25)<br>19 (12-23)<br>0.14	 0.52<br>**0.041**<br>NA
Mean Pulmonary Arterial Pressure (mmHg)<br>Pre<br>Post<br>P-Value for Change	 29 (22-39)<br>28 (22-34)<br>**<0.001**	 33 (25-46)<br>28 (21-36)<br>0.20	 0.10<br>0.94<br>NA
**Postprocedural Course**			
Intensive Care Unit Stay Duration (hours)	18.6±105.3	4.1±19.2	0.32
Hospitalization Length (days)	1 (1-5)	1 (1-6)	0.70
Discharge Home	829 (93.7)	51 (98.1)	0.36
**One-Month Medications**			
Beta Blockers	495 (68.8)	33 (67.3)	0.84
RAS Inhibitors	370 (51.8)	16 (41.0)	0.19
MRAs	149 (20.7)	6 (12.2)	0.16
Loop Diuretics	505 (70.0)	35 (71.4)	0.84

Data are presented as number (percentage), median (interquartile range), or mean±standard deviation. Figures in bold denote statistical significance.

* Improvement and normalization of the pulmonary venous flow pattern were defined as a delta S/D velocities ratio of >1 and as a postprocedural S/D velocities ratio of ≥1, respectively.

MRAs = mineralocorticoid receptor antagonists; NA = not applicable; RAS = renin-angiotensin system

Table S13. One-Month and One-Year Heart Failure and Mitral Regurgitation Indices Following First and Redo Procedures Performed at Cedars-Sinai on Different Patients.

	1-Month			1-Year		
	**First Procedure (N=902)**	**Redo Procedure (N=52)**	**P-Value**	**First Procedure (N=902)**	**Redo Procedure (N=52)**	**P-Value**
**Clinical**						
New York Heart Association Class						
I-II; P-Value vs Baseline	532 (79.8); **<0.001**	24 (58.5); **<0.001**	**0.001**	339 (77.8); **<0.001**	18 (78.3); **<0.001**	0.91
Change from Baseline (classes)	-1.5±0.8	-1.3±0.7	0.13	-1.4±0.9	-1.5±0.8	0.82
Improved (reduced) from Baseline	597 (89.5)	36 (87.8)	0.79	375 (86.0)	21 (91.3)	0.74
KCCQ12 Score						
Median (points); P-Value vs Baseline	74.31 (48.96-89.06); **<0.001**	56.25 (31.78-98.44); 1.00	0.41	76.04 (54.69-90.63); **<0.001**	93.75 (53.39-96.62); 0.32	0.29
Change from Baseline (points)	20.57 (4.43-41.15)	2.09 (-12.19-10.42)	**0.016**	27.08 (13.02-51.56)	NA	NA
Improved (increased) from Baseline	323 (82.0)	4 (50.0)	**0.043**	134 (91.2)	1 (50.0)	0.18
Furosemide-Equivalent Dose						
Median (mg/day); P-Value vs Baseline	20 (0-40); **<0.001**	40 (0-80); 0.49	**<0.001**	20 (0-20); **<0.001**	40 (20-80); **0.139**	**<0.001**
Change from Baseline (mg/day)	0 (-20-0)	0 (-20-0)	0.45	0 (-40-0)	0 (0-20)	**<0.001**
Improved (reduced) from Baseline	73 (25.8)	16 (33.3)	0.84	75 (41.0)	2 (10.5)	**0.009**
**Laboratory**						
Serum B-type Natriuretic Peptide						
Median (pg/mL); P-Value vs Baseline	646 (312-1,655); 0.51	510 (227-1,137); 0.23	0.14	397 (182-987); 0.65	524 (271-1,063); 0.13	0.37
Change from Baseline (pg/mL)	-6 (-255-205)	36 (-156-334)	0.11	-43 (-285-157)	65 (-11-331)	0.30
Improved (reduced) from Baseline	35 (50.0)	9 (31.0)	0.08	29 (53.7)	4 (30.8)	0.14

**Echocardiographic**						
Mitral Regurgitation Severity ≤Mild; P-Value vs Baseline	407 (63.7); **<0.001**	16 (43.2); **<0.001**	**0.012**	185 (53.8); **<0.001**	7 (35.0); **0.029**	0.29
Mitral Regurgitation Severity ≤Moderate; P-Value vs Baseline	606 (94.9); **<0.001**	33 (89.2); **<0.001**	0.13	320 (93.1); **<0.001**	20 (100.0); **<0.001**	0.45
Transmitral Mean Pressure Gradient						
Median (mmHg); P-Value vs Baseline	4 (3-5); **<0.001**	5 (3-7); 0.07	**0.019**	4 (3-5); **<0.001**	4 (2-5); 0.96	0.91
Change from Baseline (mmHg)	1 (0-2)	1 (-1-3)	0.37	1 (0-2)	-1 (-1-2)	0.06
Left Ventricular Ejection Fraction						
Median (%); P-Value vs Baseline	50 (30-60); **<0.001**	56 (32-61); 0.91	0.56	51 (32-60); **<0.001**	55 (30-60); 0.66	0.47
Change from Baseline (%)	-3 (-9-3)	1 (-5-5)	**0.030**	-2 (-9-4)	0 (-9-13)	0.97
Improved from Baseline	206 (33.2)	18 (51.4)	**0.027**	130 (37.8)	9 (45.0)	0.67
Left Ventricular End-Systolic Diameter						
Median (mm); P-Value vs Baseline	3.8 (3.1-4.8); 0.88	3.5 (2.7-4.4); 0.58	0.12	3.6 (2.9-4.9); **0.002**	3.8 (2.9-5.3); 0.47	0.39
Change from Baseline (mm)	0.0 (-0.4-0.4)	-0.1 (-0.4-0.3)	0.55	-0.1 (-0.5-0.4)	0.0 (-0.1-0.4)	0.22
Improved (reduced) from Baseline	288 (47.3)	16 (59.3)	0.22	181 (54.7)	6 (40.0)	0.39
Left Atrial Volume Index						
Median (cm^3/m^2); P-Value vs Baseline	54.0 (42.0-71.0); **<0.001**	61.9 (41.9-80.0); 0.39	0.41	52.0 (38.2-68.5); **<0.001**	54.0 (39.9-76.1); 0.27	0.52
Change from Baseline (cm^3/m^2)	-4.0 (-15.4-10.0)	-2.0 (-10.1-15.8)	0.43	-52.1 (-70.9-[-36.4])	-55.5 (-81.0-[-41.5])	0.13
Improved (reduced) from Baseline	296 (55.4)	8 (66.7)	0.44	753 (88.6)	27 (96.4)	0.35
Pulmonary Arterial Systolic Pressure						
Median (mmHg); P-Value vs Baseline	39 (31-49); **<0.001**	48 (37-56); 0.40	**0.011**	37 (28-49); **<0.001**	45 (35-56); 0.67	0.07
Change from Baseline (mmHg)	-6 (-15-4)	6 (-10-12)	**0.011**	-6 (-17-3)	-5 (-16-11)	0.51
Improved (reduced) from Baseline	383 (65.2)	8 (40.0)	**0.020**	214 (66.7)	7 (63.6)	0.81
Tricuspid Regurgitation Severity ≥Moderate-Severe; P-Value vs Baselne	77 (12.4); **<0.001**	14 (40.0); 0.73	**<0.001**	38 (11.0); **0.001**	6 (30.0); 1.00	0.06
**Combined**						
NYHA Class I-II and MR Severity ≤Mild; P-Value vs Baseline	340 (52.3); **<0.001**	9 (24.3); **<0.001**	**0.001**	149 (41.2); **<0.001**	5 (25.0); **<0.001**	0.29

NYHA Class I-II and MR Severity ≤Moderate; P-Value vs Baseline	484 (74.4); **<0.001**	20 (54.1); **<0.001**	**0.003**	245 (67.7); **<0.001**	14 (73.7); **<0.001**	0.98

Data are presented as number (percentage), median (interquartile range), or mean±standard deviation. Figures in bold denote statistical significance.

MR = mitral regurgitation; NA = not applicable; NYHA = New York Heart Association

Table S14. Multivariable Cox Proportional Hazard Model for the Combined Outcome of All-Cause Mortality or Heart Failure Hospitalizations at 1 Year Following Isolated, First-Time Mitral Transcatheter Edge-to-Edge Repair.

	**HR (95% CI)**	**P-Value**
Age (continuous)	0.99 (0.98-1.01)	0.31
New York Heart Association Class IV	2.09 (1.39-3.12)	**<0.001**
Acute Decompensated Heart Failure Presentation	1.89 (1.32-2.69)	**<0.001**
Atrial Fibrillation/Flutter	0.83 (0.59-1.16)	0.28
Severe Mitral Regurgitation	0.88 (0.57-1.37)	0.59
Transmitral Mean Pressure Gradient (continuous)	0.99 (0.89-1.11)	0.86
Tricuspid Regurgitation Severity ≥Moderate-Severe	1.49 (1.04-2.13)	**0.030**
TAPSE/PASP (continuous)	0.15 (0.06-0.40)	**<0.001**
Redo Mitral TEER	1.13 (0.53-2.42)	0.75
Number of Clips Deployed (continuous)	1.07 (0.86-1.33)	0.53
Use of 3rd-Generation Device	1.22 (0.84-1.76)	0.29
Non-A2P2 Clipping	1.38 (0.66-2.89)	0.40

Figures in bold denote statistical significance.

CI = confidence interval; HR = hazard ratio; PASP = pulmonary arterial systolic pressure; TAPSE = tricuspid annular plane systolic excursion; TEER = transcatheter edge-to-edge repair
